# Selective Akt Inhibitors Synergize with Tyrosine Kinase Inhibitors and Effectively Override Stroma-Associated Cytoprotection of Mutant FLT3-Positive AML Cells

**DOI:** 10.1371/journal.pone.0056473

**Published:** 2013-02-21

**Authors:** Ellen Weisberg, Qingsong Liu, Xin Zhang, Erik Nelson, Martin Sattler, Feiyang Liu, Maria Nicolais, Jianming Zhang, Constantine Mitsiades, Robert W. Smith, Richard Stone, Ilene Galinsky, Atsushi Nonami, James D. Griffin, Nathanael Gray

**Affiliations:** 1 Department of Medical Oncology, Dana Farber Cancer Institute, Harvard Medical School, Boston, Massachusetts, United States of America; 2 Department of Biological Chemistry and Molecular Pharmacology, Harvard Medical School, Boston, Massachusetts, United States of America; 3 High Magnetic Field Laboratory, Chinese Academy of Sciences, Hefei, Anhui, P.R. China; Institut national de la santé et de la recherche médicale (INSERM), France

## Abstract

**Objectives:**

Tyrosine kinase inhibitor (TKI)-treated acute myeloid leukemia (AML) patients commonly show rapid and significant peripheral blood blast cell reduction, however a marginal decrease in bone marrow blasts. This suggests a protective environment and highlights the demand for a better understanding of stromal:leukemia cell communication. As a strategy to improve clinical efficacy, we searched for novel agents capable of potentiating the stroma-diminished effects of TKI treatment of mutant FLT3-expressing cells.

**Methods:**

We designed a combinatorial high throughput drug screen using well-characterized kinase inhibitor-focused libraries to identify novel kinase inhibitors capable of overriding stromal-mediated resistance to TKIs, such as PKC412 and AC220. Standard liquid culture proliferation assays, cell cycle and apoptosis analysis, and immunoblotting were carried out with cell lines or primary AML to validate putative candidates from the screen and characterize the mechanism(s) underlying observed synergy.

**Results and Conclusions:**

Our study led to the observation of synergy between selective Akt inhibitors and FLT3 inhibitors against mutant FLT3-positive AML in either the absence or presence of stroma. Our findings are consistent with evidence that Akt activation is characteristic of mutant FLT3-transformed cells, as well as observed residual Akt activity following FLT3 inhibitor treatment. In conclusion, our study highlights the potential importance of Akt as a signaling factor in leukemia survival, and supports the use of the co-culture chemical screen to identify agents able to potentiate TKI anti-leukemia activity in a cytoprotective microenvironment.

## Introduction

Resistance to TKIs in leukemia patients presents a significant clinical challenge. As small numbers of leukemia cells have been observed to persist in the bone marrow of TKI-treated patients, despite rapid and dramatic clearance of peripheral blood blasts, there is growing interest in determining the role of the bone marrow microenvironment in the long-term survival of leukemic stem cells. Indeed, the number of existing leukemic stem cells that exhibit high survival ability on bone marrow stromal layers has proven to be a significant prognostic indicator [1]. Of relevance, we have found that media conditioned by human HS-5 stromal cells, as well as a cocktail of cytokines secreted in high concentrations by HS-5 stroma (including SCF, IL-6, IL-8, IL-11, M-CSF and GM-CSF), were able to partially protect TKI-treated chronic myeloid leukemia (CML) cells and AML cells [2], [3].

A subset of AML cells expresses a mutated form of the class III receptor tyrosine kinase FLT3 (*F*ms-*L*ike *T*yrosine kinase-3; STK-1, human *S*tem Cell *T*yrosine *K*inase-1; or FLK-2, *F*etal *L*iver *K*inase-2) [4], which has inspired the development of a number of small molecule inhibitors of mutant FLT3. However, FLT3 inhibitors tested thus far, including PKC412 (midostaurin) [5], which is in late stage (Phase III) clinical trials, and the highly potent and selective FLT3 inhibitor, AC220 (quizartinib) [6], which is in early phase clinical trials, generally at best induce partial and transient clinical responses in patients when used alone. In addition, we have found that bone marrow-derived stroma diminishes the activity of both PKC412 and AC220 [7]. There is thus a need for identification and development of novel therapies that can be effectively combined with TKIs to delay or suppress leukemia progression, override stroma-associated drug resistance, and increase patient survival.

We have recently identified the multi-targeted kinase inhibitor, dasatinib, and dasatinib-like compounds as being able to potentiate the activity of TKIs PKC412 and AC220 against mutant FLT3-expressing cells cultured in the presence of cytoprotective and cytokine-abundant stromal-conditioned media (SCM) by performing a combinatorial drug screen using the KIN001 library (Dr. Nathanael Gray) [7]. Our study also highlighted the potential of Jak inhibitors to synergize with PKC412 and AC220 as well as enhance their apoptotic activity against mutant FLT3-expressing cells cultured in the presence of SCM [7].

While the significance of stromal-derived growth factors in viability enhancement and cytoprotection of leukemic stem cells cannot be denied, not all hematologic malignancies can be rescued from programmed cell death by secreted cytokines in the absence of direct communication with the stromal cells themselves. As examples, protection of AML cells and B-lineage ALL cells from spontaneous and/or drug-induced apoptosis was observed to depend on direct bone marrow fibroblast cell:leukemic cell interaction [8]–[10]. Similarly, protection of CLL cells from apoptosis depends on adherence of these cells to bone marrow stromal layers [11], and adhesion between bone marrow stroma and myeloma cells is necessary for protection of these cells from drug-induced apoptosis [12]. Thus, the direct interaction between stromal cells and leukemic cells is important to fully understand the mechanisms driving stromal-mediated chemoresistance, as well as for identification of integral signaling molecules as potential therapeutic targets for overriding drug resistance.

To address this, we used an adherent stroma-based co-culture system, as opposed to the SCM-based system used previously, as the basis for a combinatorial drug screen designed to identify novel kinase inhibitors able to potentiate the apoptosis-inducing effects of PKC412 against adherent stroma-protected mutant FLT3-positive cells (see schematic in Figure S1, which illustrates both the adherent stroma-based screen used in this study as well as the SCM-based chemical screen [7]). In parallel to the KIN001 kinase inhibitor library, we also screened the LINCS kinase inhibitor library, which is composed of inhibitors characterized as being relatively potent and selective toward a limited range of kinase targets.

Here, we identified selective Akt inhibitors, such as MK2206, as able to effectively combine with FLT3 inhibitors, including PKC412 and AC220, against mutant FLT3-expressing cell lines or primary AML cells cultured in a cytoprotective stromal environment. This synergy occurs both in the absence as well as the presence of stroma or stromal-derived cytokines, and could thus potentially be further investigated as a therapeutic for AML as well as possibly delay/eradicate residual disease. In addition, p38 MAPK inhibitors also positively combined with PKC412 against mutant FLT3-expressing cells protected by stroma.

Our findings suggest that the combination of kinase inhibitor-enriched chemical libraries and the leukemia cell:stromal cell co-culture assay could be useful for discovery of novel therapeutic combinations for AML. This technical approach could also be employed for identification of protein kinases with potential to be exploited as novel therapeutic targets.

## Materials and Methods

### Kinase Inhibitor Focused Libraries (KIN001 and LINCS)

Two Kinase Inhibitor Focused Libraries (KIN001 and LINCS) were chosen for screening to identify single agents with little-to-no appreciable efficacy but that are able to synergize with PKC412 against the human mutant FLT3-expressing AML cell line, MOLM14-luc+, cultured in the presence of adherent HS-5 stroma. The KIN001 Library was developed by Dr. Nathanael Gray’s lab and is comprised of 188 commercially-available kinase inhibitors as well as in-house developed diverse pharmacophore-based kinase inhibitors targeting either active or inactive kinase conformations. The chemical screening concentration was 660 nM, which is the same screening concentration as was used previously when this library was used to identify kinase inhibitors able to synergize with FLT3 inhibitors in the presence of SCM [7]. The LINCS library is available from Harvard Medical School/NIH LINCS program (https://lincs.hms.harvard.edu/) and contains 202 known selective and potent kinase inhibitors.

### Cell Lines and Cell Culture

The human AML-derived, FLT3-ITD-expressing cell lines, Molm14 [13] and MV4;11 [14], were provided to us by Dr. Scott Armstrong, Dana Farber Cancer Institute, Boston, MA. MOLM14 cells were transduced with the FUW-Luc-mCherry-puro lentivirus as previously described [15]. Within the past six months, mutant FLT3 expression and integrity in this line was confirmed. The human AML-derived, FLT3-ITD-expressing cell line, MOLM-13, was obtained from DSMZ (German Resource Centre for Biological Material). MOLM-13 cells were also engineered to express luciferase fused to neomycin phosphotransferase (pMMP-LucNeo) by transduction with a VSVG-pseudotyped retrovirus (MOLM13-luc+ cells), as previously described [16]. The IL-3-dependent murine hematopoietic cell line, Ba/F3, was transduced with FLT3-ITD-containing MSCV retroviruses harboring a neomycin selectable marker, and selected for resistance to neomycin [17]. Mutant FLT3-transduced cells were selected for growth in G418 (1 mg/ml). Within the past six months, mutant FLT3 expression and integrity in this line was confirmed. The HS-5 stromal cell line was purchased from American Type Culture Collection (ATCC) (Manassas, VA, USA).

All cell lines were cultured with 5% CO_2_ at 37°C, at a concentration of 2×10^5^ to 5×10^5^/mL in RPMI (Mediatech, Inc., Herndon, VA) with 10% fetal bovine serum and supplemented with 2% L-glutamine and 1% penicillin/streptomycin.

### AML Patient Cells

Mononuclear cells were isolated from AML patients (Table S1). Mononuclear cells were isolated by density gradient centrifugation through Ficoll-Plaque Plus (Amersham Pharmacia Biotech AB, Uppsala, Sweden) at 2000 rpm for 30 minutes, followed by two washes in 1X PBS. Freeze-thawed cells were then cultured in liquid culture (DMEM, supplemented with 20% FBS) and then tested in the presence of 50% SCM with different concentrations of single and combined agents. All blood and bone marrow samples from AML patients were obtained through written consent under approval of the Dana Farber Cancer Institute Institutional Review Board. The ethics committees approved the consent procedure.

### Chemical Compounds and Biologic Reagents

PKC412 was synthesized by Novartis Pharma AG, Basel, Switzerland, and was dissolved in DMSO to obtain a 10 mM stock solution. Serial dilutions were then made, to obtain final dilutions for cellular assays with a final concentration of DMSO not exceeding 0.1%.

Dasatinib and AC220 were purchased from Haoyuan Chemexpress (Shanghai, China). KIN112 and KIN113 were developed in Dr. Gray’s lab (DFCI). KIN001 or LINCS library compounds identified in the screen as able to synergize with PKC412 in the presence of adherent HS-5 stroma were as follows: HMSL10035 (KIN001-102; Akt inhibitor); KIN001-200 (VX-702; p38 MAPK inhibitor); HMSL10168 (LG168, VX0745; p38 MAPK inhibitor).

Akt and p38 MAPK inhibitors tested to assess the significance of drugs identified in the chemical screen were as follows: HMSL10167 (SB 203580; RWJ 64809, PB 203580; p38 MAPK inhibitor); HMSL10060 (TAK-715; p38a inhibitor); HMSL10154 (AT7867; Akt inhibitor); HMSL10128 (GSK 690693; Akt inhibitor); and HMSL10057 (MK2206; Akt inhibitor) (Table S2). The selective Akt inhibitors used in our study are very well-characterized research tools that have been widely used in different contexts (for inhibitor characteristics and background, please see Table S2).

### Cell Proliferation, Viability and Cell Cycle Analysis

The trypan blue exclusion assay (for quantifying cells prior to seeding), Annexin-V-Fluos Staining Kit (Boehringer Mannheim, Indianapolis, IN) (for apoptosis), and cell cycle analysis were carried out as previously described [5]. Due to technical convenience, SCM was used instead of adherent stroma for the apoptosis and cell cycle assays. The Cell Titer Glo assay (Promega, Madison, WI) (for proliferation) was used for proliferation studies, and carried out according to manufacturer instructions.

### Antibodies and Immunoblotting

For analysis of phospho- and total Akt and phospho- and total GSK3β, MOLM14-luc+ cells were treated with drugs for two hours before they were collected at 1100 rpm for 8 minutes. Cells were lysed using M-PER lysis buffer (Pierce) supplemented with phosphatase inhibitors and protease inhibitors (Roche) according to the manufacturers’ instructions. Equivalent amounts of proteins were loaded. For analysis of phospho- and total STAT5, phospho- and total S6K, and phospho- and total MAPK, immunoblotting was carried out as previously described [5].

The following primary antibodies were purchased from Cell Signaling Technology (Danvers, MA): Anti-phospho-Akt (T308), anti-phospho-Akt (S473), and anti-Akt. The following primary antibodies were purchased from Sigma-Aldrich (St Louis, MO): Anti-tubulin, anti-GSK3β, and anti-phospho-GSK3β. All antibodies were used at 1∶1000 dilution, except for anti-tubulin, which was used at 1∶5000. The following primary antibodies (for data shown in supporting data section) were purchased from Cell Signaling Technology (Danvers, MA): Phospho-STAT5 (Tyr 694) (C11C5) (rabbit, #9359), used at 1∶500, total STAT5 (rabbit, #9363), used at 1∶1000, phospho-p70 S6K (Thr389) (1A5) (mouse, #9206), used at 1∶300, total p70 S6K (49D7) (rabbit, #2708), used at 1∶1000, phospho-p44/42 MAPK (Erk1/2) (Thr202/Tyr204) (rabbit, #9101), used at 1∶1000, total p44/42 MAPK (Erk1/2) (3A7) (mouse, #9107), used at 1∶1000. HRP conjugated secondary antibodies were purchased from Promega and were used at a dilution of 1∶5000.

### Drug Combination Studies

For drug combination studies, single agents were added simultaneously at fixed ratios to mutant FLT3-expressing cells cultured in the presence of adherent HS-5 stroma, 50–80% SCM, or RPMI+10% FBS. Cell viability was determined using the Trypan Blue exclusion assay to quantify cells for cell seeding, and Cell Titer Glo for proliferation studies. Cell viability was expressed as the function of growth affected (FA) drug-treated versus control cells; data were analyzed by Calcusyn software (Biosoft, Ferguson, MO and Cambridge, UK), using the Chou-Talalay method [18]. The combination index = [D]_1_ [D_x_]_1_+ [D]_2_/[D_x_]_2_, where [D]_1_ and [D]_2_ are the concentrations required by each drug in combination to achieve the same effect as concentrations [D_x_]_1_ and [D_x_]_2_ of each drug alone. Values less than one indicate synergy, whereas values greater than one indicate antagonism. Calcusyn combination indices can be interpreted as follows: CI <0.1 indicate very strong synergism; values 0.1–0.3 indicate strong synergism; values 0.3–0.7 indicate synergism; values 0.7–0.85 indicate moderate synergism; values 0.85–0.90 indicate slight synergism; values 0.9–1.1 indicate nearly additive effects; values 1.10–1.20 indicate slight antagonism; values 1.20–1.45 indicate moderate antagonism; values 1.45–3.3 indicate antagonism; values 3.3–10 indicate strong antagonism; values >10 indicate very strong antagonism. Note: For some experiments, namely those in which there was no observed single agent activity due to stromal protection, combination indices could not be reliably calculated using the Calcusyn software.

### Human Adherent Stroma Validation Experiments

HS-5 human stromal cells (10,000/well) were determined in a pilot study to be sufficient for maximum cytoprotection of PKC412-treated MOLM14-luc+ cells (Figure S2). Stromal cells were seeded 24 hours in advance of seeding MOLM14-luc+ cells (4000/well), followed by drug treatments. The Bright Glo assay (Promega, Madison, WI) was performed for co-culture assays to selectively measure leukemia cell viability and was carried out according to manufacturer’s instructions.

## Results

### Chemical Screen Identification of Inhibitors able to Potentiate Effects of PKC412 against Mutant FLT3-expressing Cells co-cultured with Adherent Human Stromal Cells

In the present study, which is a direct and intentional extension of our previous work [7], we set out to compare the use of SCM and adherent stroma as the basis for a chemical screen geared toward identification of drugs capable of overriding drug resistance due to stromal influences. Specifically, we conducted an unbiased combinatorial screen of 188 compounds comprising the KIN001 chemical library in an attempt to identify kinase inhibitors able to synergize with PKC412 against mutant FLT3-positive cells co-cultured with adherent stroma. Similar to previous findings using HS-5 SCM [7], three dual Src/Abl inhibitors- dasatinib, KIN112, and KIN113- were identified as being able to positively combine with PKC412 against MOLM14-luc+ co-cultured with adherent HS-5 stroma cells as a replacement for SCM (Figure S3). In addition to confirming previously published findings, these results also validate the use of either SCM or adherent stroma as part of a chemical screen approach to identify agents able to override drug resistance due to a cytoprotective microenvironment.

**Figure 1:**
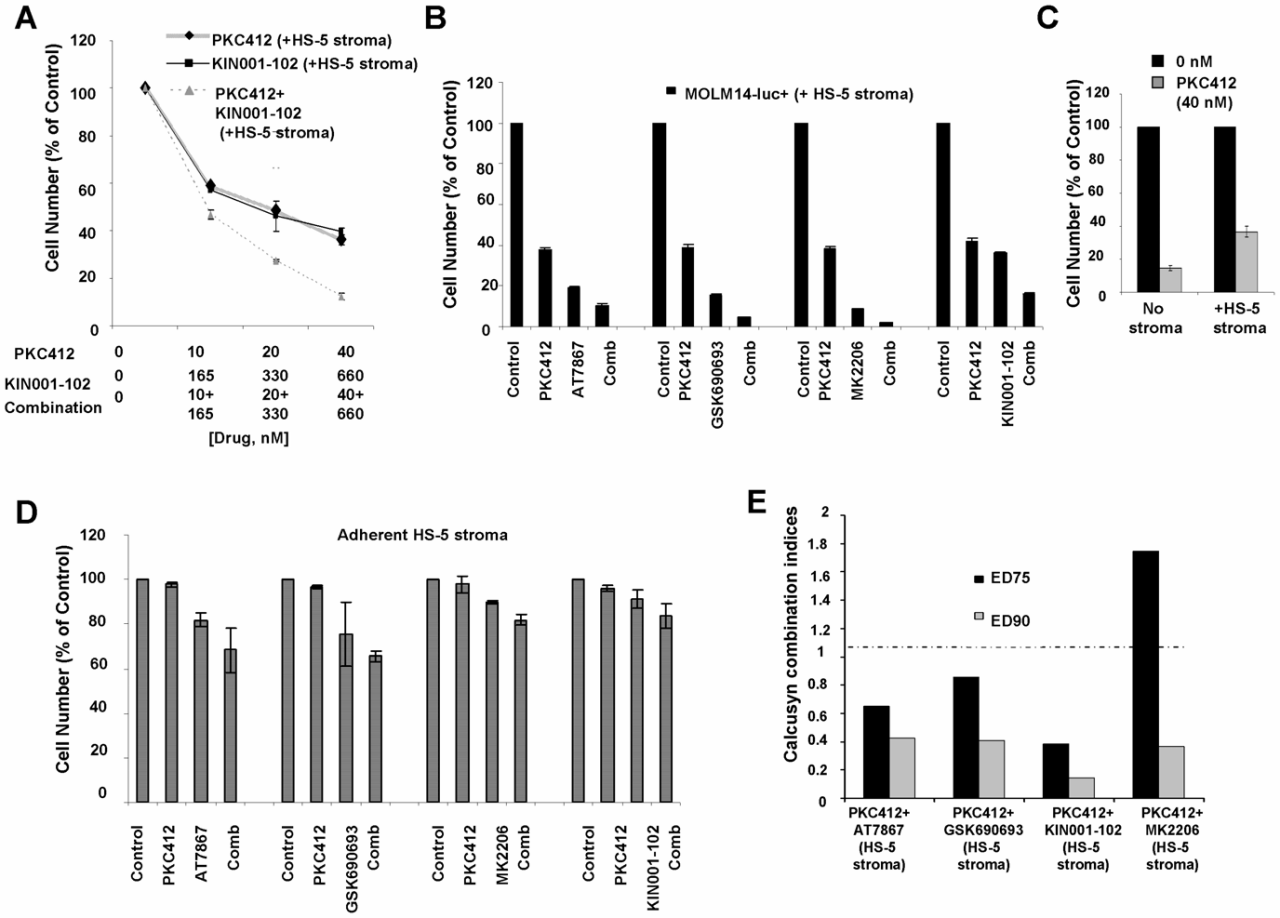
Selective inhibitors of AKT positively combine with PKC412 in the presence of adherent HS-5 stroma against MOLM14-luc+ cells. (A) Approximately two-day proliferation study performed with MOLM14-luc+ cells cultured in the presence of adherent HS-5 stroma testing the combination of PKC412 and KIN001-102 versus each agent alone. (B) MOLM14-luc+ cells cultured in the presence of adherent HS-5 stroma for approximately two days: PKC412 (40 nM)+/− Akt inhibitors (660 nM). (C) Approximately two-day PKC412 treatment of MOLM14-luc+ cells cultured in the absence and presence of human stroma. (D) Approximately two-day treatment of adherent HS-5 stroma: PKC412 (40 nM) +/− Akt inhibitors (660 nM). (E) Calcusyn combination indices derived from 4-point concentration proliferation experiments. The cut-off for nearly additive effects (C.I.: 1.1) is marked by a dashed line.

**Figure 2:**
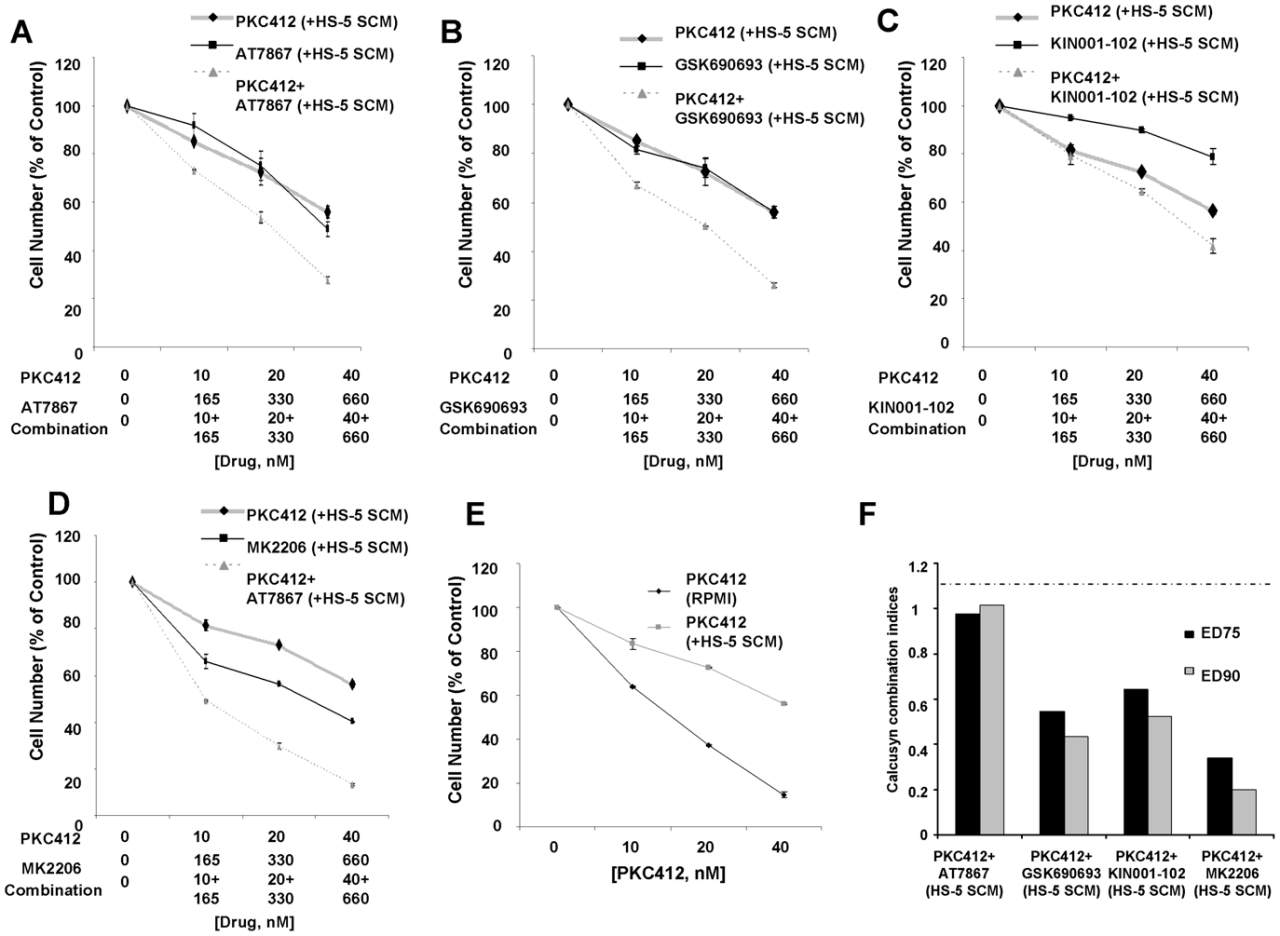
Selective inhibitors of AKT positively combine with PKC412 in the presence of SCM against MOLM14-luc+ cells. (A–D) Approximately two-day proliferation studies performed with selective AKT inhibitors in combination with PKC412 in the presence of HS-5 SCM. (E) Approximately two-day PKC412 treatment of MOLM14-luc+ cells cultured in the absence or presence of HS-5 SCM (n = 2). (F) Calcusyn combination indices derived from the 4-point concentration proliferation experiments shown in A-D. The cut-off for nearly additive effects (C.I.: 1.1) is marked by a dashed line.

We also identified library-derived inhibitors of major signaling pathways, including the allosteric Akt inhibitor, KIN001-102, as able to positively combine with PKC412 against adherent stroma-protected mutant FLT3-expressing cells (Figure 1A). In order to validate whether or not Akt as a therapeutic target was important for the observed higher percentage of killing of stromal-protected cells when used in combination with PKC412, we tested a panel of selective Akt inhibitor analogs against MOLM14-luc+ cells under the same co-culture conditions. Similar to KIN001-102, the selective Akt inhibitors, AT7867, GSK690693, and MK2206 positively combined with PKC412 against MOLM14-luc+ cells cultured in either the presence of adherent HS-5 stroma (Figure 1B, C) or HS-5 SCM (Figure 2), with combination indices at ED75-ED90 suggestive of synergy (Figures 1 and 2).

To further validate the co-culture model for the combination drug screen, we investigated the effects of single agents and combination treatments on adherent stromal cells. This would establish whether or not stromal cell killing (and hence removal of the source of protective secreted cytokines) played a role in the observed synergy between PKC412 and Akt inhibitors. To address this, selective Akt inhibitors were tested against adherent HS-5 stroma directly. Compared to inhibitor effects against MOLM14-luc+ cells, inhibitor activity against adherent stroma was considerably weaker (Figure 1B, D). In addition, whereas PKC412 (40 nM) and selective Akt inhibitors (660 nM) were highly effective alone and combined against Ba/F3 cells expressing mutant FLT3, the same drugs at the same concentrations displayed little-to-no appreciable effects against parental Ba/F3 cells and displayed little activity in the presence of 15% WEHI as a source of IL-3 (Figure S4). These data, taken together, suggest that drug activity observed against mutant FLT3-expressing cells is due to on-target effects.

In addition to Akt inhibitors, positive hits from the chemical library screens also included inhibitors of p38 MAPK inhibitors, which positively combined with PKC412 against MOLM14-luc+ cells cultured in the presence of adherent HS-5 stroma (Figure S5). However, the ability of p38 MAPK inhibitors to positively combine with PKC412 was substantially diminished when mutant FLT3-expressing cells were cultured in the presence of HS-5 SCM as opposed to adherent stroma (Figure S5). There exists the possibility that high levels of stromal-secreted cytokines may negatively influence the synergizing potential of p38 MAPK inhibitors with FLT3 inhibitors. Hence, Akt inhibitors may be superior in terms of their overall combination potential and general ability to override stromal-mediated drug resistance and were therefore our main focus in this study.

### FLT3 Inhibitor and Akt Inhibitor Combination Effects on Cell Cycle Progression and Apoptosis of Stroma-protected AML Cells

Synergy observed between PKC412 and KIN001-102 against MOLM14-luc+ cells cultured in the presence of 50% SCM correlated with induction of apoptosis, as drug combination-treated cells showed the highest percentages of apoptotic cells (Table 1). An increase in the G1 population was observed for MOLM14-luc+ cells cultured for 24 hr in the presence of 50% SCM and treated with PKC412 alone (approximately 86% of cells were in G1/G0). Combination treatments led to approximately 89% of cells in G1/G0 (Table 1), which is a comparatively small increase in percentage. In contrast, compared to PKC412 alone, combination treatment of MOLM14-luc+ cells for 48 hr resulted in substantially increased apoptosis (PKC412 alone: 27.1% apoptosis, versus combination treatments: 41.3%-48.9% apoptosis) (Table 1 and Figure S6 Part I and II). Stromal protection was evidenced by the fact that PKC412 treatment of MOLM14-luc+ cells in RPMI+10% FBS led to 47% viable cells (Table 2 and Figure S7 Part I and II), whereas PKC412 treatment of MOLM14-luc+ cells in the presence of SCM led to 71% viable cells (Table 1 and Figures S6 Part I and II). These results suggest that induction of apoptosis, more than cell cycle arrest, contributes to the observed synergy between PKC412 and KIN001-102 against mutant FLT3-expressing cells cultured in a cytoprotective stromal environment.

**Table 1:** Effects of PKC412 and KIN001-102, alone and combined, on MOLM14-luc+ cell cycle progression (following 24 hours of treatment) and apoptosis (following 48 hours of treatment) when cells are cultured in the presence of 50% HS-5 SCM.

	Cell Cycle Progression			Cell Viability		
MOLM14-luc+Treatment	% G1/G0	% G2M	% S	% Viable	% Apoptotic	% Necrotic
DMSO Control	67.95	6.270	25.78	84.70	14.00	1.400
PKC412 (40 nM)	86.24	3.070	10.68	71.00	27.10	2.000
KIN001-102 (165 nM)	71.27	5.090	23.64	81.50	17.30	1.200
KIN001-102 (330 nM)	73.81	4.750	21.44	80.90	17.70	1.400
KIN001-102 (660 nM)	77.79	4.910	17.30	77.80	20.30	2.000
PKC412 (40 nM)+KIN001-102 (165 nM)	88.87	2.980	8.150	57.30	41.30	1.400
PKC412 (40 nM)+ KIN001-102 (330 nM)	89.47	2.310	8.220	52.10	45.70	2.200
PKC412 (40 nM)+ KIN001-102 (660 NM)	89.11	3.880	7.010	47.30	48.90	3.900

Details of the assays used for these studies are provided in the Materials and Methods section.

**Table 2:** Effects of PKC412 and KIN001-102, alone and combined, on MOLM14-luc+ cell cycle progression (following 24 hours of treatment) and apoptosis (following 48 hours of treatment) when cells are cultured in the presence of RPMI+10% FBS.

	Cell Cycle Progression			Cell Viability		
MOLM14-luc+ Treatment	% G1/G0	% G2M	% S	% Viable	% Apoptotic	% Necrotic
DMSO Control	56.10	9.320	34.58	92.50	6.900	0.600
PKC412 (40 nM)	86.94	1.50	11.56	46.90	52.70	0.400
KIN001-102 (165 nM)	59.60	7.360	33.04	92.00	7.800	0.300
KIN001-102 (330 nM)	61.03	9.010	29.96	91.80	8.000	0.200
KIN001-102 (660 nM)	61.93	7.030	31.05	90.90	8.800	0.300
PKC412 (40 nM)+KIN001-102 (165 nM)	85.22	3.430	11.35	31.50	68.30	0.200
PKC412 (40 nM)+ KIN001-102 (330 nM)	86.92	2.760	10.32	18.10	81.70	0.100
PKC412 (40 nM)+ KIN001-102 (660 NM)	81.11	3.160	15.73	16.90	82.80	0.300

Details of the assays used for these studies are provided in the Materials and Methods section.

**Figure 3:**
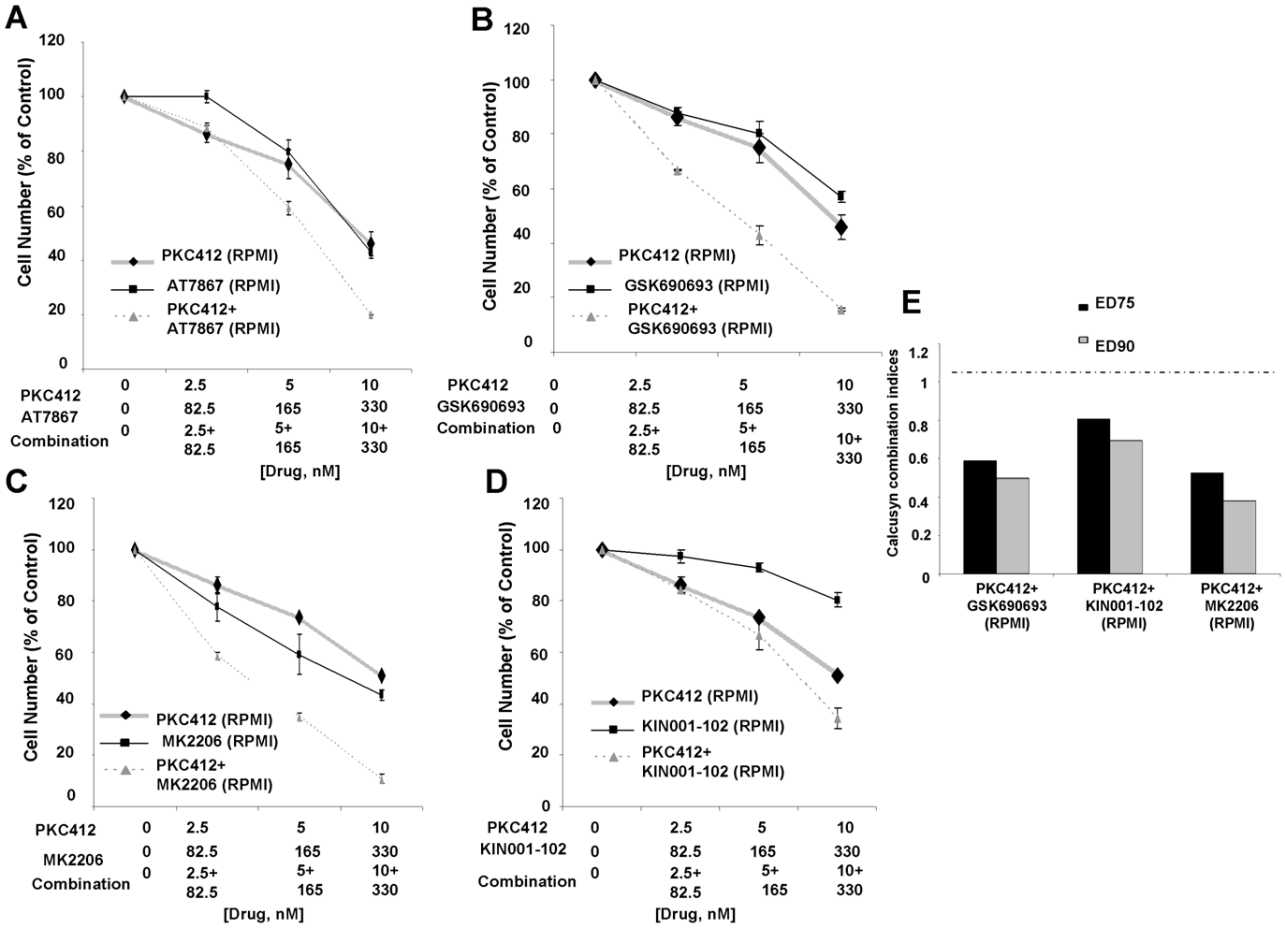
Selective inhibitors of AKT positively combine with PKC412 in RPMI+10% FBS against MOLM14-luc+ cells. (A–D) Approximately two-day proliferation studies performed with selective AKT inhibitors in combination with PKC412 in RPMI+10% FBS. (E) Calcusyn combination indices. The cut-off for nearly additive effects (C.I.: 1.1) is marked by a dashed line.

**Figure 4:**
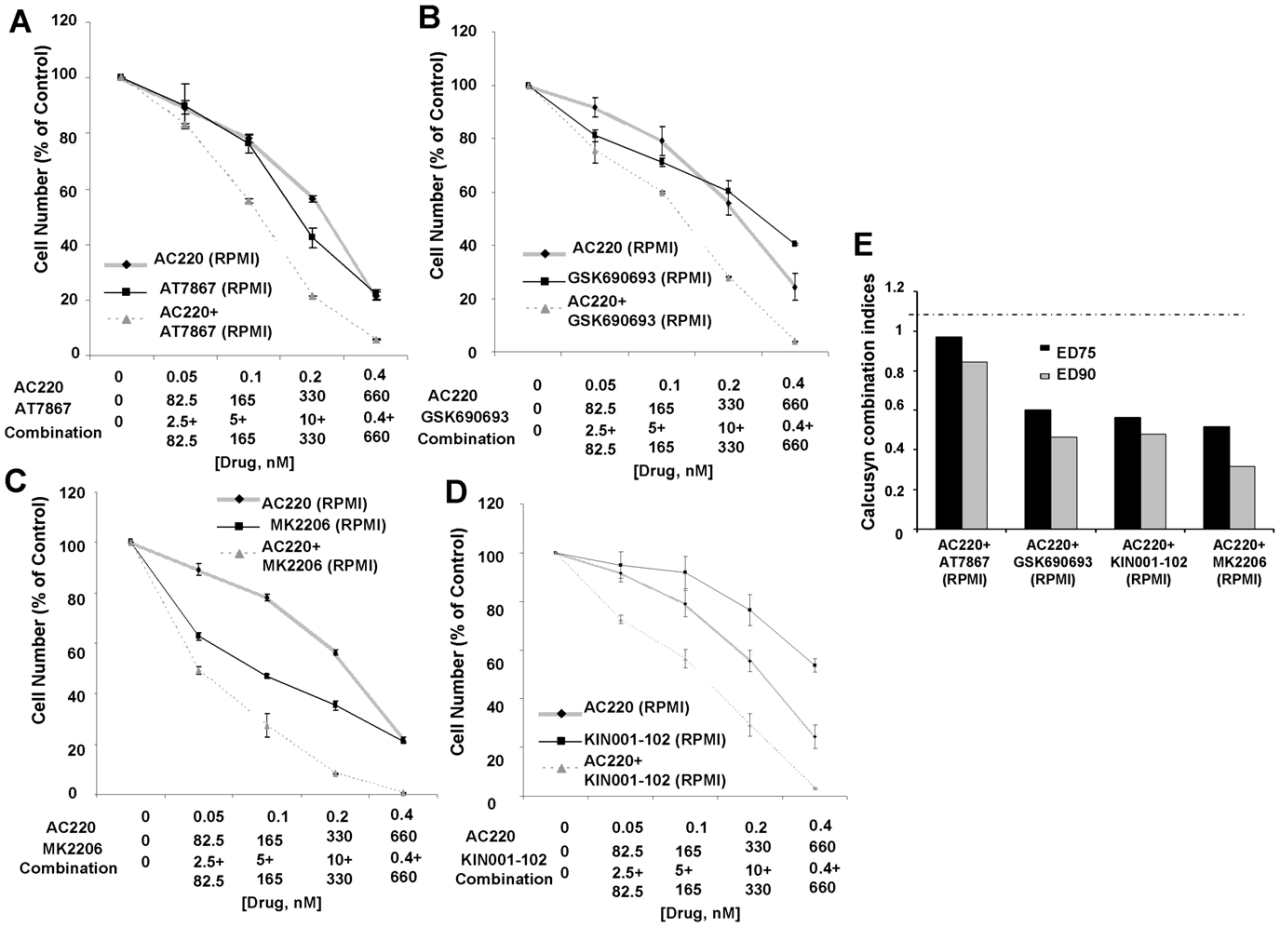
Selective inhibitors of AKT positively combine with AC220 in RPMI+10% FBS against MOLM14-luc+ cells. (A–D) Approximately two-day proliferation studies performed with selective AKT inhibitors in combination with AC220 in RPMI+10% FBS. (E) Calcusyn combination indices. The cut-off for nearly additive effects (C.I.: 1.1) is marked by a dashed line.

Synergy was observed between PKC412 and selective Akt inhibitors against MOLM14-luc+ cells cultured in the presence of RPMI+10% FBS (Figure 3). Synergy was also observed between selective Akt inhibitors and the highly potent and selective FLT3 inhibitor, AC220, against mutant FLT3-positive leukemia cells cultured in RPMI+10% FBS (Figure 4). The ability of selective Akt inhibitors to positively combine with FLT3 inhibitors against mutant FLT3-positive AML cells in the presence of RPMI+10% FBS correlated well with induction of apoptosis, as the combination of PKC412 and KIN001-102 showed the highest percentages of cell killing as compared to single agent effects (Table 2 and Figure S7 Part I and II). After 48 hrs in RPMI+10% FBS, however, the combination of PKC412 and KIN001-102 did not lead to greater G1 arrest than PKC412 alone (40 nM) for MOLM14-luc+ cells (Table 2).

**Figure 5:**
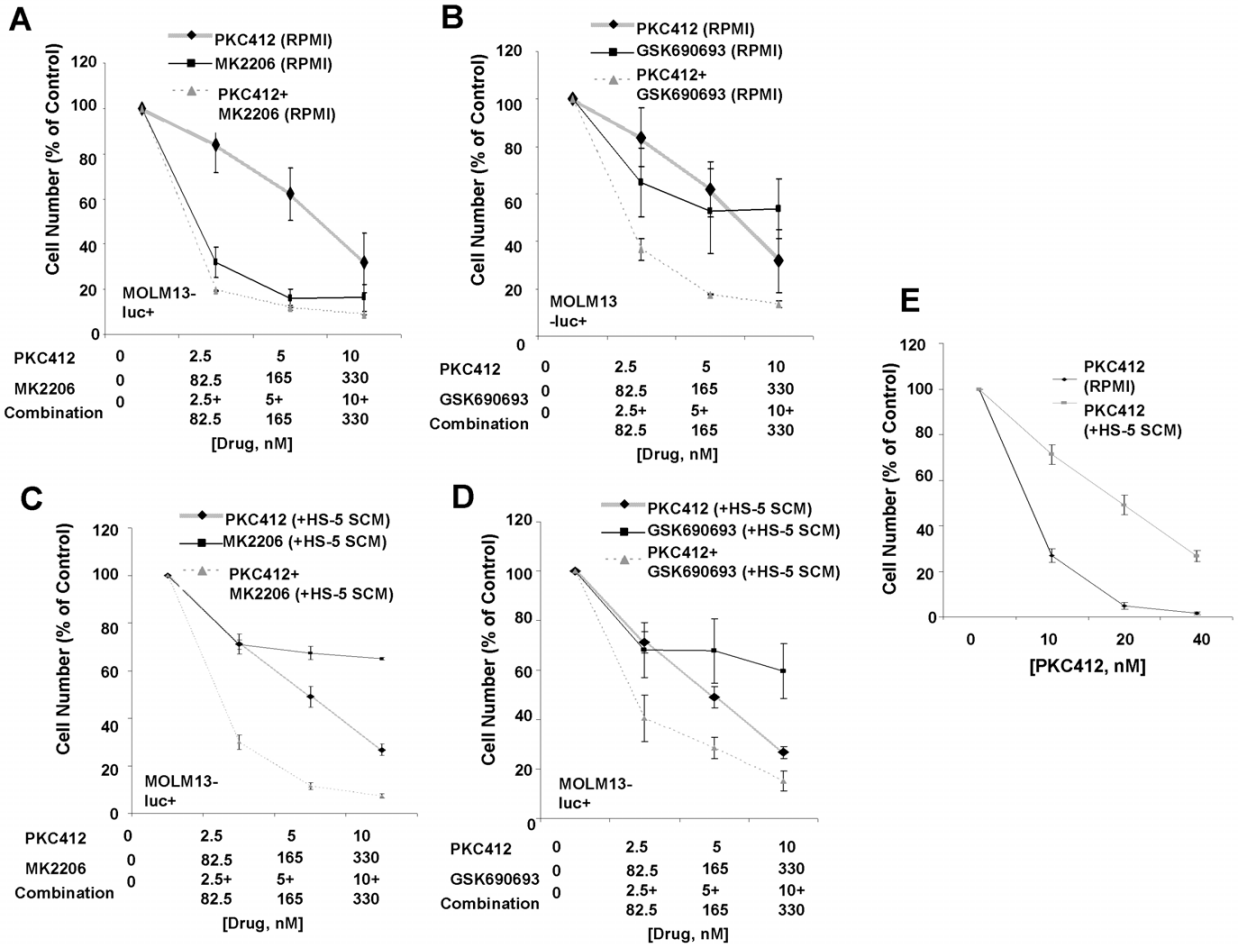
Selective inhibitors of AKT positively combine with PKC412 in the absence and presence of HS-5 SCM against MOLM13-luc+ cells. (A–B) Approximately two-day proliferation studies performed with selective AKT inhibitors in combination with PKC412 in the presence of RPMI+10% FBS. (C–D) Approximately two-day proliferation studies performed with selective AKT inhibitors in combination with PKC412 in 80% HS-5 SCM. (E) Treatment of MOLM13-luc+ cells with PKC412 in either RPMI+10% FBS or 80% HS-5 SCM.

**Figure 6:**
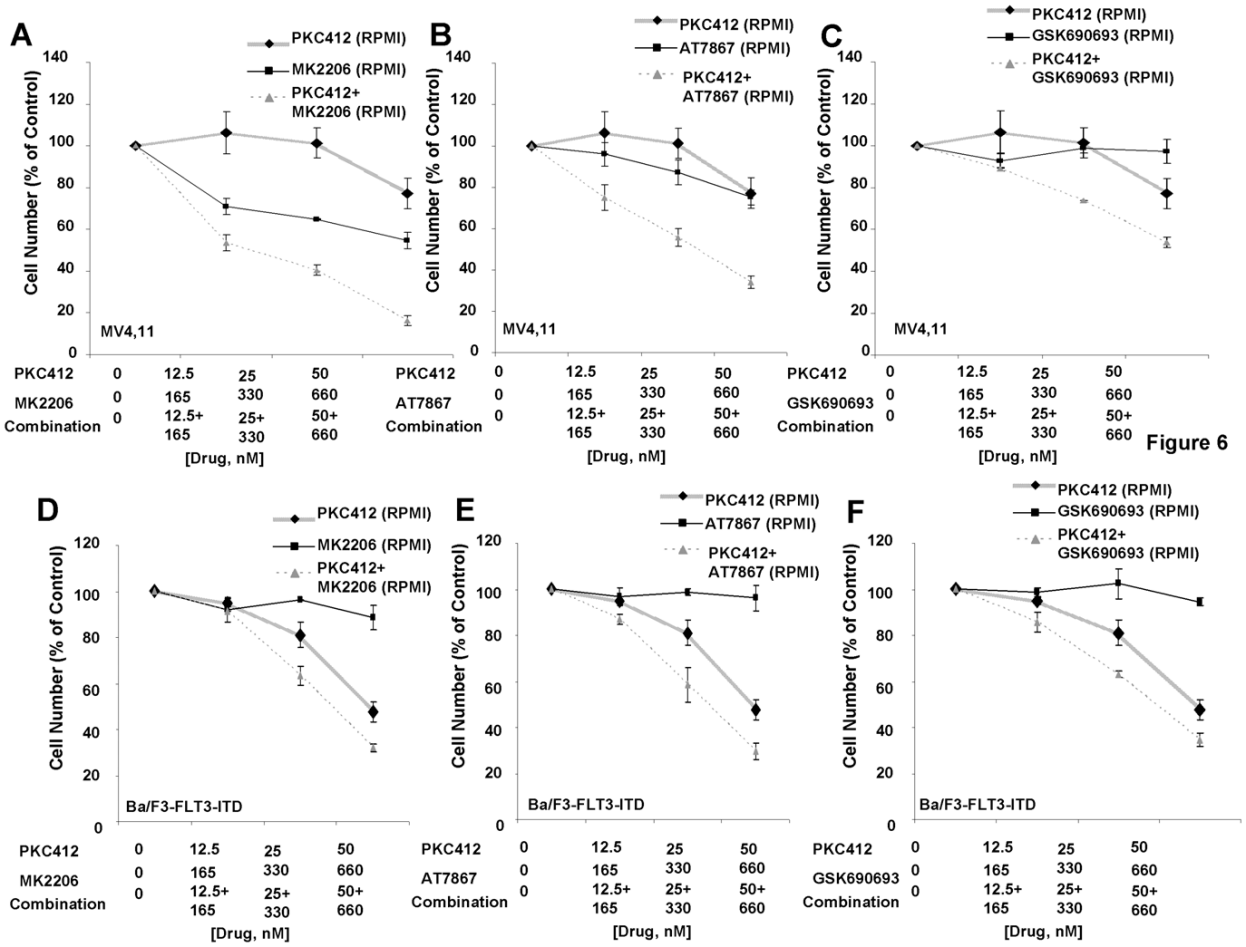
Selective inhibitors of AKT positively combine with PKC412 in RPMI+10% FBS against MV4,11 and Ba/F3-FLT3-ITD cells. (A–C) Approximately two-day proliferation studies performed with selective AKT inhibitors in combination with PKC412 in RPMI+10% FBS against MV4,11 cells. (D–F) Approximately two-day proliferation studies performed with selective AKT inhibitors in combination with PKC412 in RPMI+10% FBS against Ba/F3-FLT3-ITD cells.

Synergy between the selective Akt inhibitors and PKC412 was additionally observed in Ba/F3-FLT3-ITD cells and the two mutant FLT3-expressing human cell lines, MOLM13-luc+ and MV4,11, cultured in the presence of RPMI+10% FBS (Figures 5 and 6, Table 3, Figure S8). Partial protection of PKC412-treated MOLM13-luc+ cells was observed when cells were cultured in the presence of HS-5 SCM (Figure 5), and combinations of selective Akt inhibitors and PKC412 were synergistic against MOLM13-luc+ cells cultured in the presence of HS-5 SCM (Figure 5, Table 3).

**Table 3:** Calcusyn software-derived combination indices.

Drug Combination	Cell Line	ED25	ED50	ED75	ED90
PKC412+MK2206	Ba/F3-FLT3-ITD	0.73063	0.71787	0.70932	0.70100
PKC412+AT7867	Ba/F3-FLT3-ITD	0.62418	0.65624	0.68996	0.72540
PKC412+GSK690693	Ba/F3-FLT3-ITD	0.64287	0.72479	0.81965	0.92849
PKC412+MK2206(2-day)	MOLM13	0.54469	0.52206	0.55200	0.71660
PKC412+MK2206(3-day)	MOLM13	0.15758	0.22911	0.34332	0.55817
PKC412+AT7867(2-day)	MOLM13	0.95116	1.02639	1.11056	1.20483
PKC412+AT7867(3-day)	MOLM13	0.76889	0.26391	0.40983	0.64279
PKC412+GSK690693(2-day)	MOLM13	0.97412	0.29241	0.33779	0.56141
PKC412+GSK690693(3-day)	MOLM13	1.11399	0.79787	0.62777	0.57590
PKC412+MK2206(SCM)	MOLM13	0.75681	0.23956	0.26180	0.29207
PKC412+GSK690693(SCM)	MOLM13	0.91433	0.39599	0.51463	0.72551
PKC412+MK2206	MV4,11	0.84669	0.39562	0.43775	0.76686
PKC412+AT7867	MV4,11	0.45640	0.67869	1.08529	1.84914

Data shown here correspond to dose-response curves shown in Figures 5 and 6 and Figure S8. Interpretation of combination indices is provided in the Materials and Methods section.

### Phospho-Akt Mediates Synergy Observed between Allosteric Akt Inhibitor, KIN001-102, and PKC412

**Figure 7:**
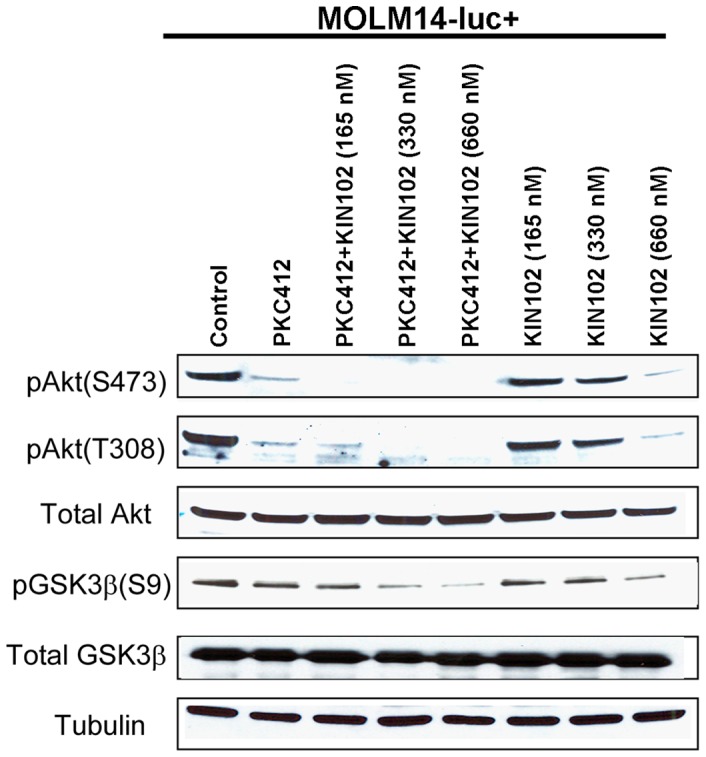
Phospho-Akt mediates synergy observed between allosteric Akt inhibitor, KIN001-102, and PKC412. Immunoblots of protein lysates prepared from MOLM14-luc+ cells treated for 2 hours with PKC412 (40 nM), KIN001-102 (165, 330, 660 nM), or a combination of the two agents in the presence of 50% SCM. Data shown are representative of two independent experiments in which similar results were achieved.

In order to verify observed combination effects in terms of signaling, we examined the phosphorylation status of Akt following either single agent treatment or combined drug treatment. Immunoblots demonstrated that pAKT levels were inhibited to a greater extent in MOLM14-luc+ cells cultured in the presence of 50% HS-5 SCM upon treatment with a combination of KIN001-102 and PKC412, as compared to either drug alone (Figure 7). The expression of GSK3β was additionally investigated as GSK3β is a direct substrate of protein kinase Akt. These results suggest that Akt activity is critical for maintaining stromal cytoprotection under these conditions. In contrast to the robust drug combination effect observed against phospho-Akt (S473) at lower concentrations of KIN102, there was no apparent combination effect observed between PKC412 (40 nM) and KIN102 (165 nM) against phospho-S6K for MOLM14-luc+ cells cultured in the presence of SCM (data not shown).

Similarly, no significant changes were observed in expression of phosphorylated S6K in MOLM14-luc+ cells cultured in RPMI+10% FBS and treated for 1 hr with PKC412 (5 nM)+/−MK2206 (165 nM) (Figure S9). Each agent was tested at respective concentrations that led to a substantial combination effect in proliferation studies (Figure 3). PKC412 alone and PKC412 combined with MK2206 decreased phosphorylation of STAT5 to similar extents in these cells, and no significant changes were observed in expression of phosphorylated MAPK between single agent-treated and drug combination-treated cells (Figure S9).

### Ability of Akt Inhibitors to Potentiate the Activity of PKC412 or AC220 against Primary AML Patient Cells Cultured in the Presence of Cytoprotective SCM

**Figure 8:**
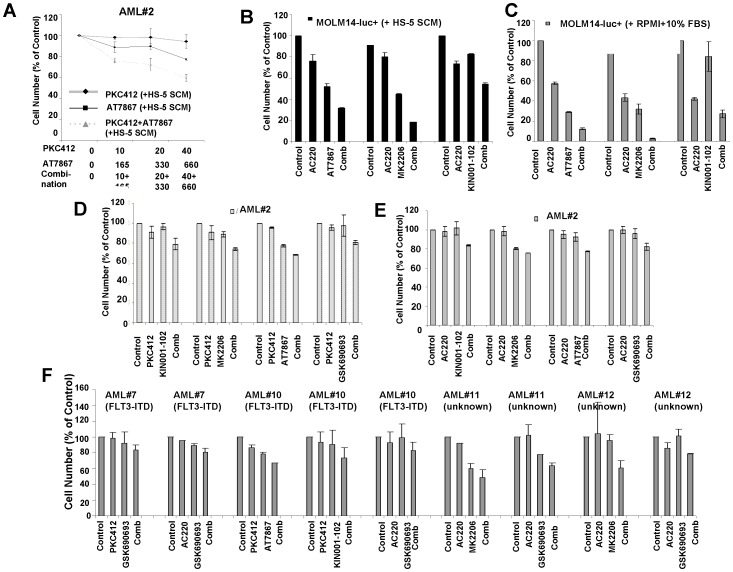
Ability of Akt inhibitors to positively combine with PKC412 or AC220 against AML patient samples in the presence of cytoprotective SCM. (A) Approximately two-day proliferation study performed with a selective Akt inhibitor in combination with PKC412 in the presence of HS-5 SCM against mutant FLT3-positive AML#2. (B) Approximately two-day combination studies: AC220 (0.4 nM) +/− selective AKT inhibitors (660 nM) against MOLM14-luc+ cells in the presence of 50% HS-5 SCM. (C) Approximately two-day combination studies: AC220 (0.4 nM) +/− selective AKT inhibitors (660 nM) against MOLM14-luc+ cells in the presence of RPMI+10% FBS. (D) Approximately two-day combination studies: PKC412 (40 nM)+/− selective AKT inhibitors (660 nM) against primary AML patient cells in the presence of 50% HS-5 SCM. (E) Approximately two-day combination studies: AC220 (0.4 nM) +/− selective AKT inhibitors (660 nM) against primary AML patient cells in the presence of 50% SCM. (F) Ability of Akt inhibitors to positively combine with PKC412 or AC220 against primary AML cells in the presence of cytoprotective SCM. Patient information is provided in Table S1.

In order to validate the stromal cell co-culture screening model, we tested the lead drugs from the screening on primary AML patient cells cultured in the presence of cytoprotective HS-5-derived SCM. Several of these samples were confirmed to express FLT3-ITD. Combination studies between PKC412 and KIN001-102, AT7867, MK2206, and GSK690693, respectively, showed the highest degree of cell killing in combination-treated, SCM-protected primary AML cells as compared to any single agent (a representative dose-response experiment for a highly drug-resistant AML patient sample (#2) is shown in Figure 8A). Analysis of the combinatorial effect with Calcusyn revealed synergy (ED75∶0.39202, ED90∶0.55992) for PKC412+AT7867 against mutant FLT3-positive AML#2.

Given the multiple targets of PKC412, we investigated the ability of AC220, which has a very high selectivity index, to combine with Akt inhibitors. A positive combination effect was observed between AC220 and selective inhibitors of Akt against MOLM14-luc+ cells cultured in the presence of HS-5-derived SCM (Figure 8B). AC220 similarly combined positively with Akt inhibitors against MOLM14-luc+ cells cultured in RPMI+10% FBS with combination indices at ED75 and ED90 suggestive of synergy (Figure 8C).

Selective Akt inhibitors were thus also tested for their ability to potentiate the effects of AC220, as compared to PKC412, against primary AML patient cells cultured in the presence of HS-5-derived SCM. As shown in Figures 8D–F, PKC412 and AC220, which were tested in parallel, resulted in the highest degree of patient cell killing when either drug was used in combination with Akt inhibitors as compared to any single agent against a panel of primary AML samples. Of note, all drug treatments were performed in the presence of SCM, which provides cytoprotective cytokines and dampens inhibitor efficacy.

## Discussion

Previous studies of ours suggest that TKI-dependent combination therapy likely represents a potentially useful approach to counteracting both intrinsic and stroma-associated drug resistance in leukemia patients [7], [19], [20], [21]. With the recent discovery of numerous FLT3 inhibitor-responsive serine/threonine and tyrosine phosphorylation sites uncovered in primary AML patient bone marrow samples [22], identification of protein kinase inhibitors that are able to enhance the potency of FLT3 inhibitors makes intuitive sense.

Here, selective inhibitors targeting kinases involved in PI3K/Akt and Ras/MEK/MAPK signaling were identified in a chemical screen as synergizing with PKC412 against mutant FLT3-expressing cells in the presence of adherent stroma. Akt inhibitors synergized with FLT3 inhibitors in the presence of either SCM or adherent stroma, as compared to p38 MAPK inhibitors, which synergized with FLT3 inhibitors only in the presence of adherent stroma. One possibility for this may be traced to the nature of stromal protection by SCM, characterized by highly concentrated levels of stromal-derived cytokines.

Of relevance, studies have implicated Akt- and MAPK-mediated signaling in stromal enhancement of leukemia cell viability. For instance, co-culture of leukemia cells and bone marrow-derived stroma has been shown to lead to activation of the MAPK/ERK pathway and integrin-linked kinase (ILK), which phosphorylates Akt [22]. ILK/Akt is likely critical for leukemia cell survival in bone marrow, and thus inhibitors of ILK have been proposed as an approach to simultaneously target both leukemia cells and leukemia-activated stromal cells [23]. Additionally, p38 MAPK activation has been found to play a role in stroma-dependent survival of B-CLL cells [24] and ALL cells [25].

In addition, continuous FLT3 inhibitor treatment leads to the development of drug-resistant cells characterized by constitutive activation of parallel downstream PI3K/Akt and/or Ras/MEK/MAPK signaling pathways, which is believed to compensate for the loss of FLT3 activity in terms of survival and growth [26]. In support of this, constitutive activation of ERK/Akt/STAT pathways has been observed in AML despite small molecule inhibition of FLT3-ITD activity, suggesting that optimal treatment of AML may require FLT3 inhibition combined with inhibition of additional signaling pathways [27]. Dual inhibition of FLT3 and Akt-mediated signaling, such as that conferred by the multiple kinase inhibitor, KP372-1, has indeed been found to inhibit primary AML cell growth with minimal effect on normal progenitor cells [28].

Consistent with our results is the finding that Akt, p38MAPK, and Erk activation correlates with development of resistance of BCR-ABL-positive acute lymphoblastic leukemia (ALL) to nilotinib plus the farnesyltransferase inhibitor lonafarnib [28]. Inhibitors of Akt and Erk combined respectively with nilotinib diminished resistance. In contrast to our findings, however, inhibition of p38 MAPK in this study increased TKI (nilotinib) resistance [29].

Importantly, we observed synergy between selective Akt inhibitors and FLT3 inhibitors in the absence of stroma as well as its presence, suggesting that this synergy is not specific to leukemia cells growing in a cytoprotective microenvironment. Of significance, there are reports that have been and that are continuing to be published that support the potential clinical importance of inhibiting components of major signaling pathways in combination with TKIs as a way to treat AML.

The identification of Akt and p38 MAPK inhibitors as able to potentiate the effects of FLT3 inhibitors is at least in part attributable to the use of the LINCS library to identify comparatively “clean” kinase inhibitors, in contrast to the chemical library screened previously [7], which included a number of multi-kinase inhibitors such as dasatinib. A chemical library composed of relatively selective inhibitors offers a significant technical advantage in that it translates into easier elucidation of mechanism of inhibition by a single agent and synergy between agents as the drug targets are more well-defined and easier to validate.

Our in vitro findings with cell lines and primary patient samples, which closely reflect the genetic heterogeneity amongst AML patients, warrant further testing and validation in preclinical models of progressive leukemia and minimal residual disease. In vivo models that reflect stromal cell interactions, however, are fairly complex and are beyond the scope of this study. We are planning to address these questions in future studies.

In conclusion, selective inhibition of kinases such as Akt in combination with FLT3 inhibitors in mutant FLT3-positive AML patients may represent a novel approach to improving treatment effects and patient survival. Findings presented here may provide novel options for adjunctive therapy.

## Supporting Information

Figure S1 **Schematic of kinase inhibitor-focused chemical screen approaches.** Stromal-conditioned media (SCM)- or adherent stroma-based chemical libraries are used to identify agents that are able to potentiate the effects of FLT3 inhibitors against mutant FLT3-expressing cells cultured in a cytoprotective microenvironment.
(TIF)

Figure S2 **Co-culture pilot study.** Approximately 1500 MOLM14-luc+ cells were tested in a two-day assay in the presence and absence of HS-5 stroma seeded at 10,000 cells/well, 20,000 cells/well, and 40,000 cells/well.
(TIF)

Figure S3 **Coculture chemical screen identification of KIN001 library compound, dasatinib, and dasatinib-like compounds, KIN112 and KIN113, as able to synergize with PKC412 in the presence of adherent HS-5 stroma against MOLM14-luc+ cells.** (A–C) Approximately two-day assays, validating the combination potential of the KIN001 co-culture chemical screen identified agents (dasatinib, KIN112, KIN113) to synergize with PKC412 against MOLM14-luc+ cells in the presence of adherent HS-5 stroma. Approximately 5000 MOLM14-luc+ cells were seeded/well; approximately 10,000 HS-5 stromal cells were seeded/well. (D) PKC412 treatment of MOLM14-luc+ cells cultured in the absence or presence of adherent HS-5 stroma (n = 2). (E) Calcusyn combination indices. The cut-off for nearly additive effects (C.I.: 1.1) is marked by a dashed line.
(TIF)

Figure S4 **Treatment of parental Ba/F3 cells and Ba/F3-FLT3-ITD cells with PKC412, alone and in combination with selective inhibitors of Akt.** (A) Approximately three-day drug treatment of parental Ba/F3 cells cultured in the presence of IL-3 and Ba/F3-FLT3-ITD cells cultured in the absence of IL-3. (B) Approximately three-day drug treatment of Ba/F3-FLT3-ITD cells cultured in the presence of IL-3. PKC412 was used at 40 nM and selective AKT inhibitors were each used at 660 nM.
(TIF)

Figure S5 **Selective inhibitors of p38 MAPK positively combine with PKC412 against MOLM14-luc+ cells cultured in the presence of adherent HS-5 stroma, however not HS-5 SCM.** Calcusyn combination indices. The cut-off for nearly additive effects (C.I.: 1.1) is marked by a dashed line.
(TIF)

Figure S6 **Part 1.** Annexin/pi staining corresponding to data shown in Table 1: Effects of PKC412 (40 nM) and KIN001-102 (165, 330, 660 nM), alone and combined, on MOLM14-luc+ cell apoptosis (following 48 hours of treatment) when cells are cultured in the presence of 50% HS-5 SCM. Cells labeled “dying” are in early apoptotic phase, and cells labeled “apoptotic” are in late apoptotic phase. **Part 2.** Quantitative values corresponding to data shown in Figure S6 (part 1): Effects of PKC412 (40 nM) and KIN001-102 (165, 330, 660 nM), alone and combined, on MOLM14-luc+ cell apoptosis (following 48 hours of treatment) when cells are cultured in the presence of 50% HS-5 SCM. Cells labeled “dying” are in early apoptotic phase, and cells labeled “apoptotic” are in late apoptotic phase.
(DOC)

Figure S7 **Part 1.** Annexin/pi staining corresponding to data shown in Table 2: Effects of PKC412 (40 nM) and KIN001-102 (165, 330, 660 nM), alone and combined, on MOLM14-luc+ cell apoptosis (following 48 hours of treatment) when cells are cultured in the presence of RPMI+10% FBS. Cells labeled “dying” are in early apoptotic phase, and cells labeled “apoptotic” are in late apoptotic phase. **Part 2.** Quantitative values corresponding to data shown in Figure S7 (part 1): Effects of PKC412 (40 nM) and KIN001-102 (165, 330, 660 nM), alone and combined, on MOLM14-luc+ cell apoptosis (following 48 hours of treatment) when cells are cultured in the presence of RPMI+10% FBS. Cells labeled “dying” are in early apoptotic phase, and cells labeled “apoptotic” are in late apoptotic phase.
(DOC)

Figure S8 **Selective inhibitors of AKT positively combine with PKC412 in RPMI+10% FBS against MOLM13-luc+ cells.** (A–C) Approximately three-day proliferation studies performed with selective AKT inhibitors in combination with PKC412 in RPMI+10% FBS against MOLM13-luc+ cells.
(TIF)

Figure S9 **Investigation of phosphorylation of signaling molecules downstream of FLT3.** Immunoblots of protein lysates prepared from MOLM14-luc+ cells treated for 1 hour with PKC412 (5 nM), MK2206 (165 nM), or a combination of the two agents in RPMI+10% FBS.
(TIF)

Table S1 **Patient sample information.** Patients shown here were cultured in the presence of 50% HS-5 SCM, and treated with different combinations of kinase inhibitors. *Patient information for AML patients 2 and 7 has been previously published (Weisberg et al, 2012a, Leukemia).
(DOC)

Table S2 **Selective AKT and p38 MAPK inhibitors.** *Hirai H, Soontome H, Nakatsuru Y, Miyama K, Taguchi S, Tsujioka K et al. MK-2206, an allosteric Akt inhibitor, enhances antitumor efficacy by standard chemotherapeutic agents or molecular targeted drugs in vitro and in vivo. Mol Cancer Ther 2010;9∶1956-67. **Levy DS, Kahana JA, Kumar R. AKT inhibitor, GSK690693, induces growth inhibition and apoptosis in acute lymphoblastic leukemia cell lines. Blood 2009;113∶1723-9. ***Grimshaw KM, Hunter LJ, Yap TA, Heaton SP, Walton MI, Woodhead SJ, et al. AT7867 is a potent and oral inhibitor of AKT and p70 S6 kinase that induces pharmacodynamic changes and inhibits human tumor xenograft growth. Mol Cancer Ther 2010;9∶1100-10.
(DOC)
