# Supplementary figures and images for: Selective Akt Inhibitors Synergize with Tyrosine Kinase Inhibitors and Effectively Override Stroma-Associated Cytoprotection of Mutant FLT3-Positive AML Cells
Source: PLoS One. 2013 Feb 21;8(2):e56473. doi: 10.1371/journal.pone.0056473 (PMC3578845; doi:10.1371/journal.pone.0056473)

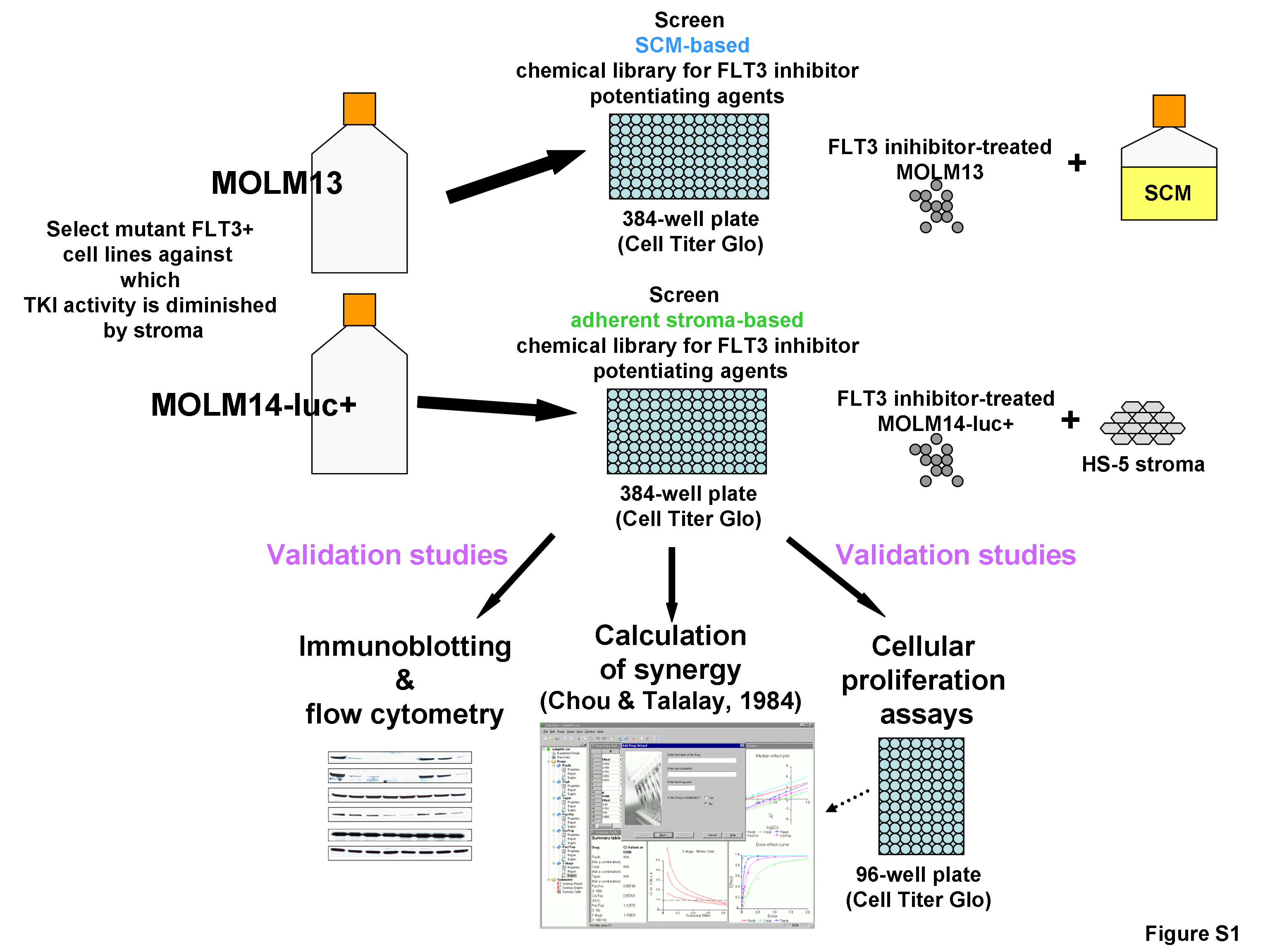

Supplement: Figure S1 — Schematic of kinase inhibitor-focused chemical screen approaches. Stromal-conditioned media (SCM)- or adherent stroma-based chemical libraries are used to identify agents that are able to potentiate the effects of FLT3 inhibitors against mutant FLT3-expressing cells cultured in a cytoprotective microenvironment. (TIF) [file pone.0056473.s001.tif]

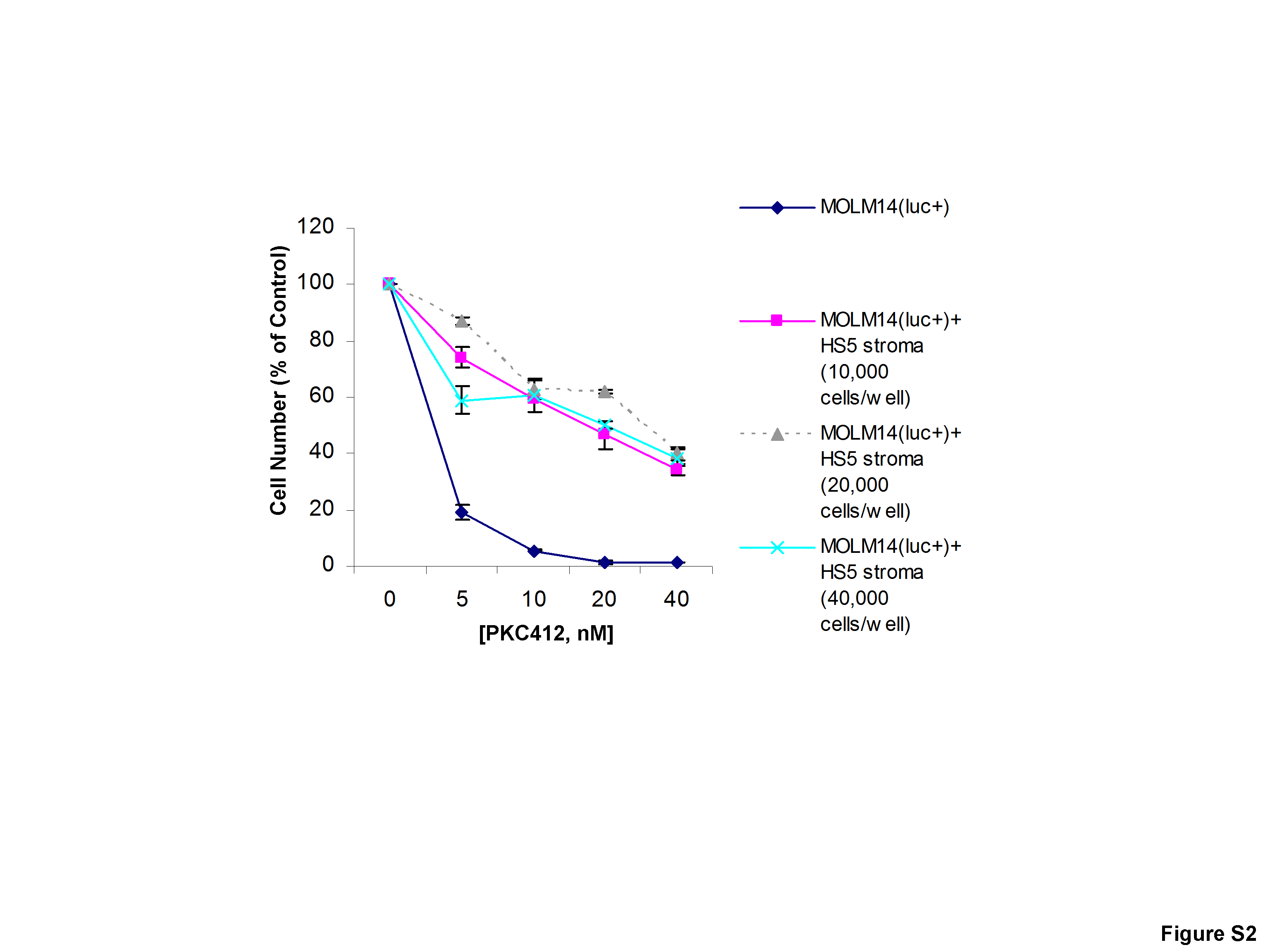

Supplement: Figure S2 — Co-culture pilot study. Approximately 1500 MOLM14-luc+ cells were tested in a two-day assay in the presence and absence of HS-5 stroma seeded at 10,000 cells/well, 20,000 cells/well, and 40,000 cells/well. (TIF) [file pone.0056473.s002.tif]

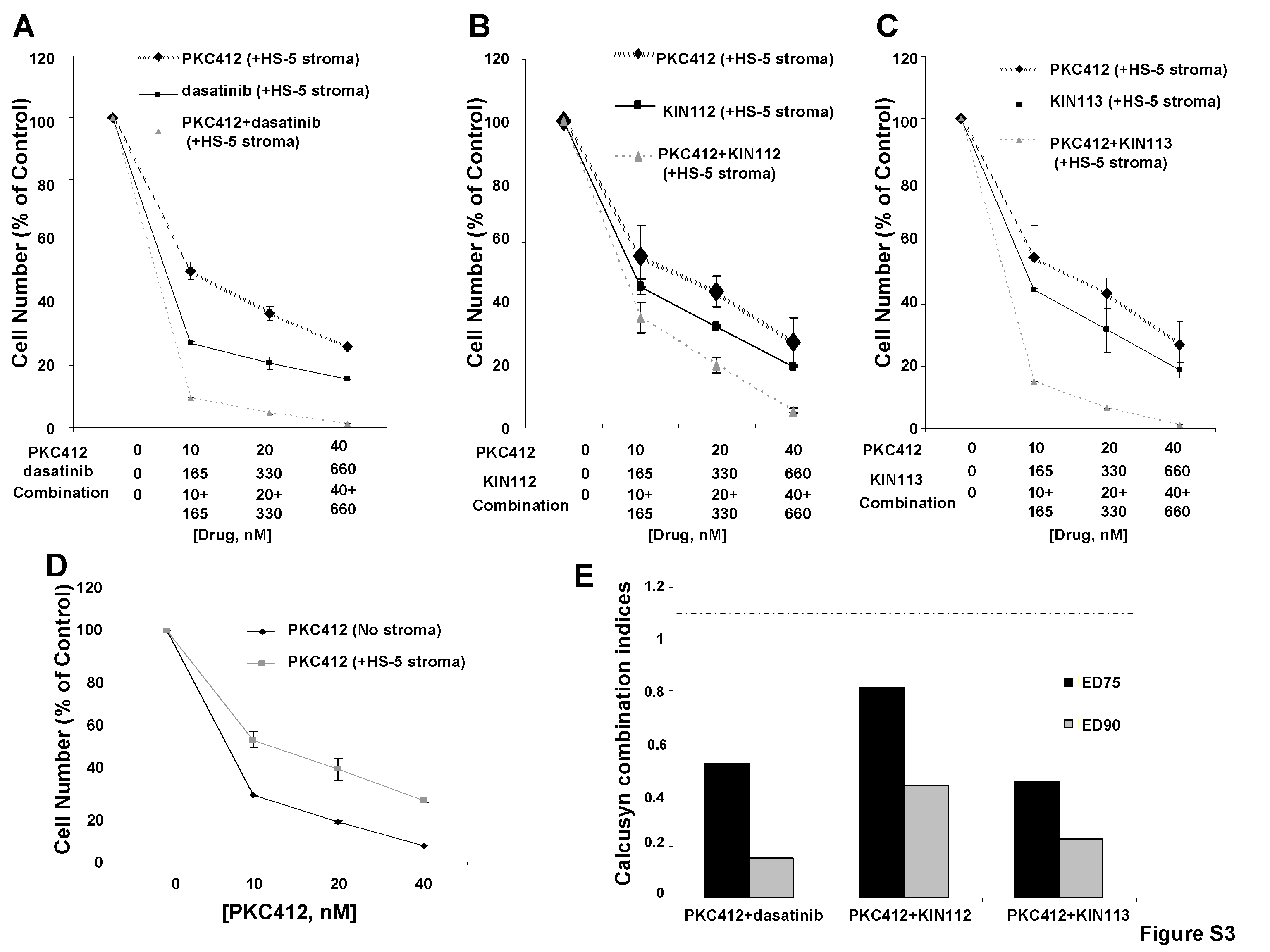

Supplement: Figure S3 — Coculture chemical screen identification of KIN001 library compound, dasatinib, and dasatinib-like compounds, KIN112 and KIN113, as able to synergize with PKC412 in the presence of adherent HS-5 stroma against MOLM14-luc+ cells. (A–C) Approximately two-day assays, validating the combination potential of the KIN001 co-culture chemical screen identified agents (dasatinib, KIN112, KIN113) to synergize with PKC412 against MOLM14-luc+ cells in the presence of adherent HS-5 stroma. Approximately 5000 MOLM14-luc+ cells were seeded/well; approximately 10,000 HS-5 stromal cells were seeded/well. (D) PKC412 treatment of MOLM14-luc+ cells cultured in the absence or presence of adherent HS-5 stroma (n = 2). (E) Calcusyn combination indices. The cut-off for nearly additive effects (C.I.: 1.1) is marked by a dashed line. (TIF) [file pone.0056473.s003.tif]

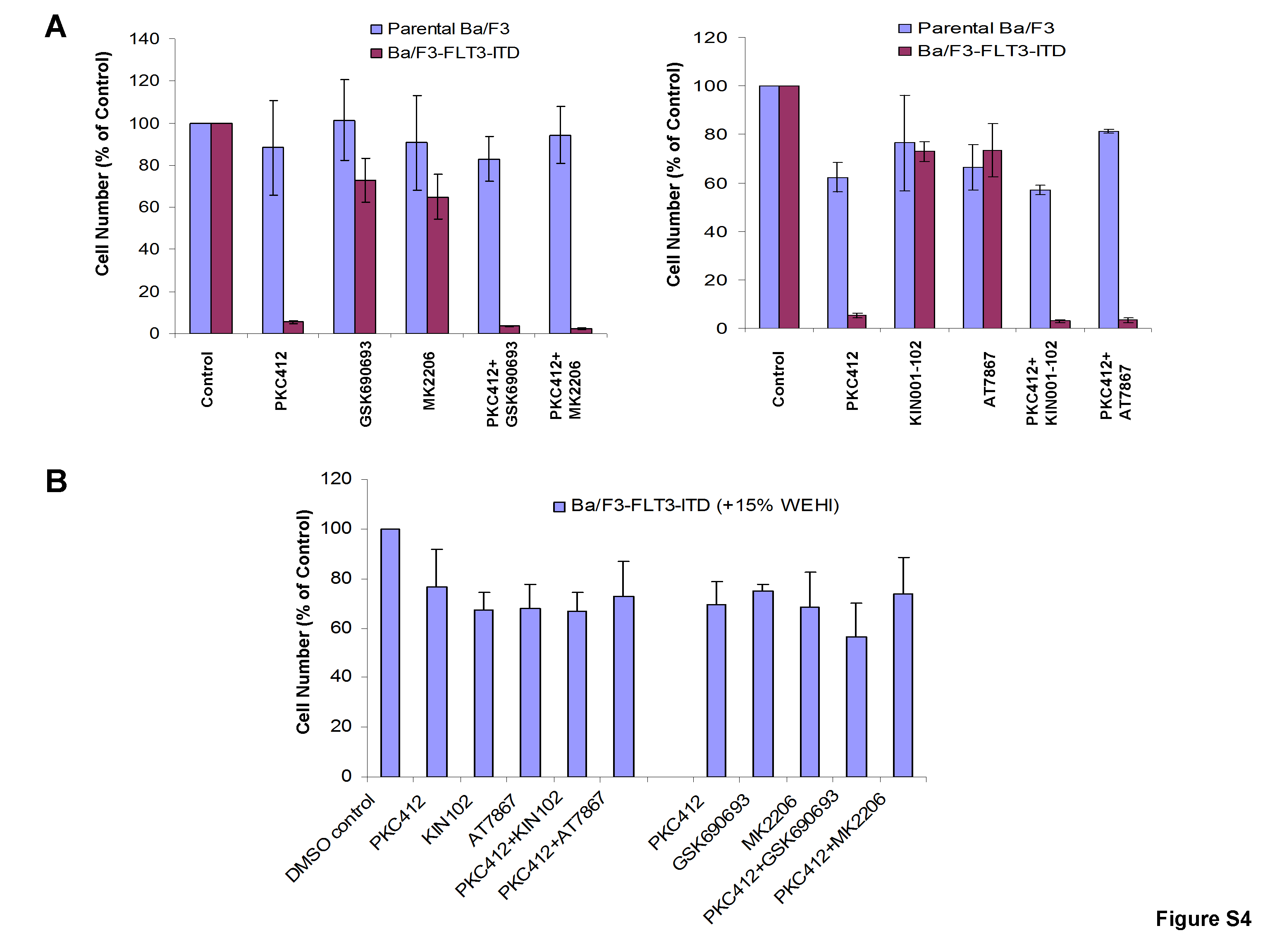

Supplement: Figure S4 — Treatment of parental Ba/F3 cells and Ba/F3-FLT3-ITD cells with PKC412, alone and in combination with selective inhibitors of Akt. (A) Approximately three-day drug treatment of parental Ba/F3 cells cultured in the presence of IL-3 and Ba/F3-FLT3-ITD cells cultured in the absence of IL-3. (B) Approximately three-day drug treatment of Ba/F3-FLT3-ITD cells cultured in the presence of IL-3. PKC412 was used at 40 nM and selective AKT inhibitors were each used at 660 nM. (TIF) [file pone.0056473.s004.tif]

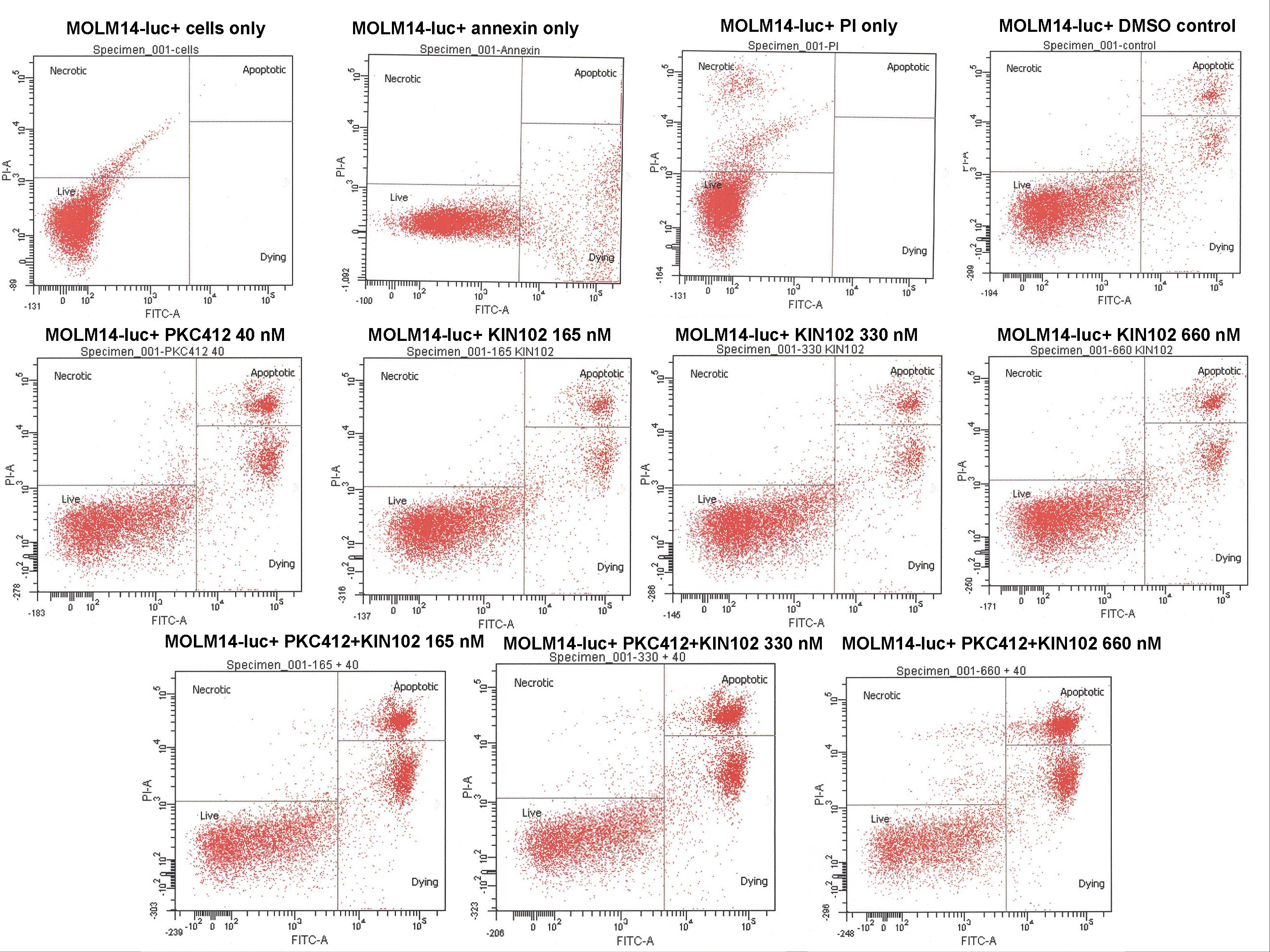


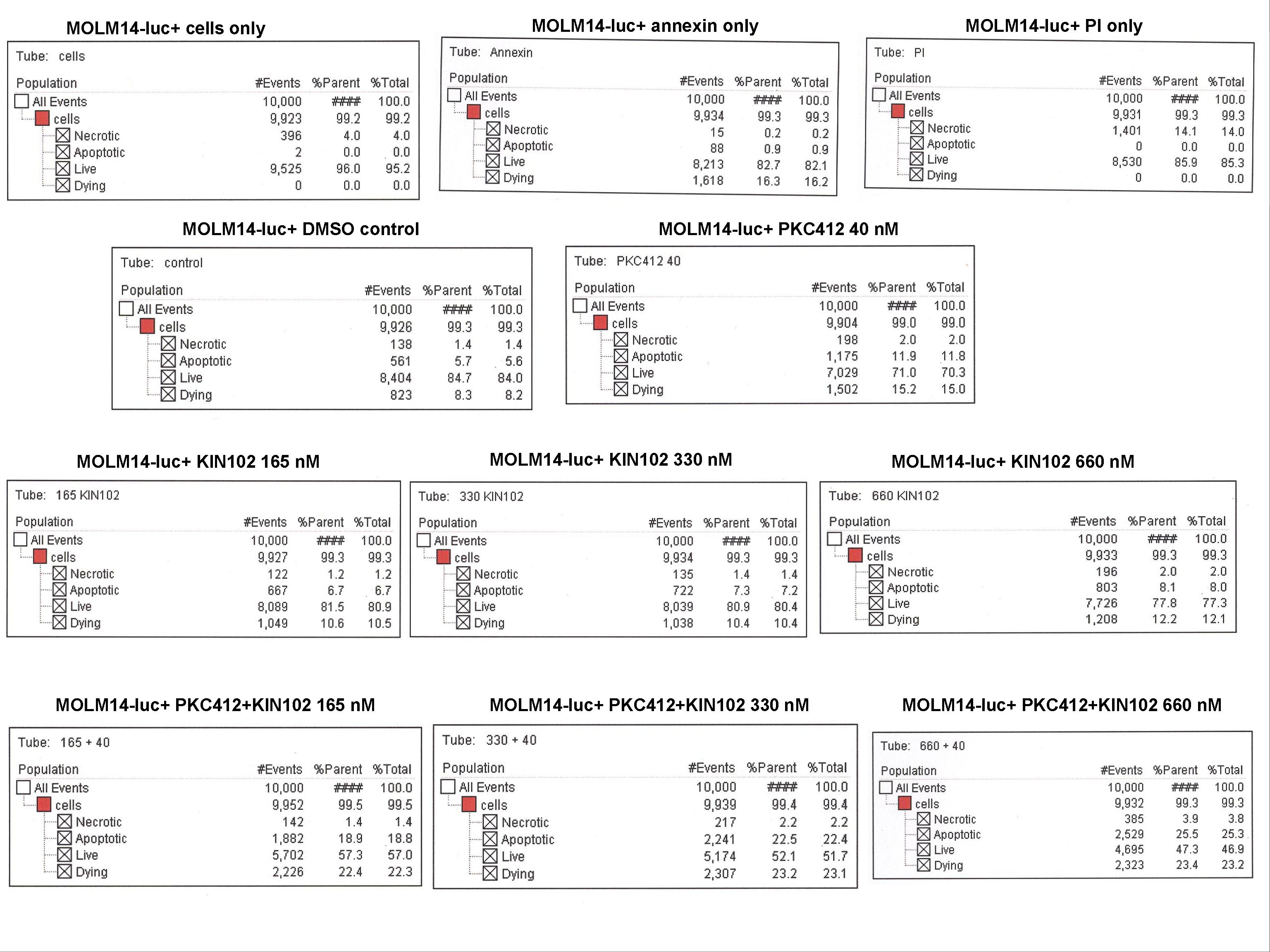


**Figure S6**

Supplement: Figure S6 — Part 1. Annexin/pi staining corresponding to data shown in Table 1: Effects of PKC412 (40 nM) and KIN001-102 (165, 330, 660 nM), alone and combined, on MOLM14-luc+ cell apoptosis (following 48 hours of treatment) when cells are cultured in the presence of 50% HS-5 SCM. Cells labeled “dying” are in early apoptotic phase, and cells labeled “apoptotic” are in late apoptotic phase. Part 2. Quantitative values corresponding to data shown in Figure S6 (part 1): Effects of PKC412 (40 nM) and KIN001-102 (165, 330, 660 nM), alone and combined, on MOLM14-luc+ cell apoptosis (following 48 hours of treatment) when cells are cultured in the presence of 50% HS-5 SCM. Cells labeled “dying” are in early apoptotic phase, and cells labeled “apoptotic” are in late apoptotic phase. (DOC) [file pone.0056473.s006.doc]

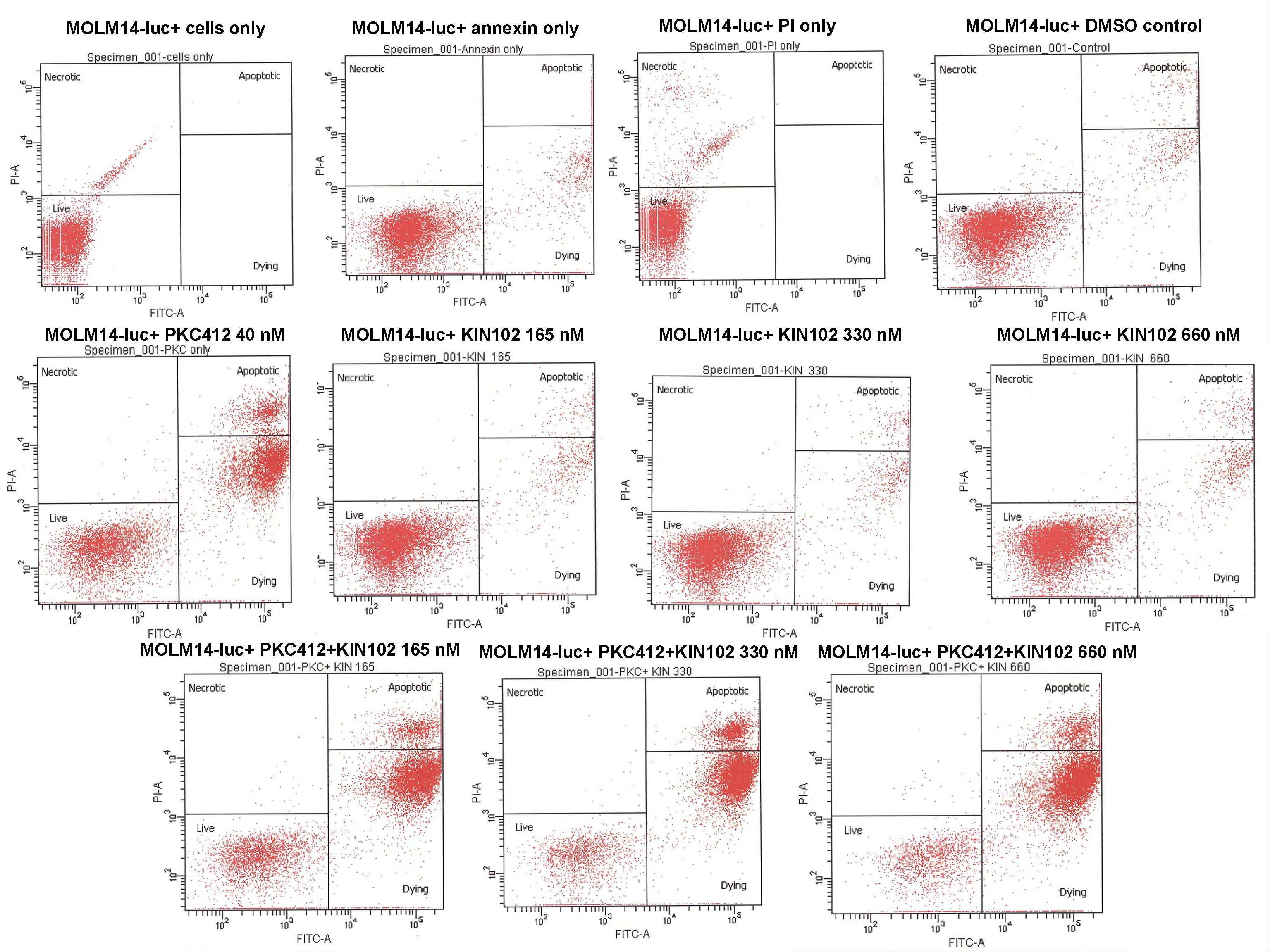


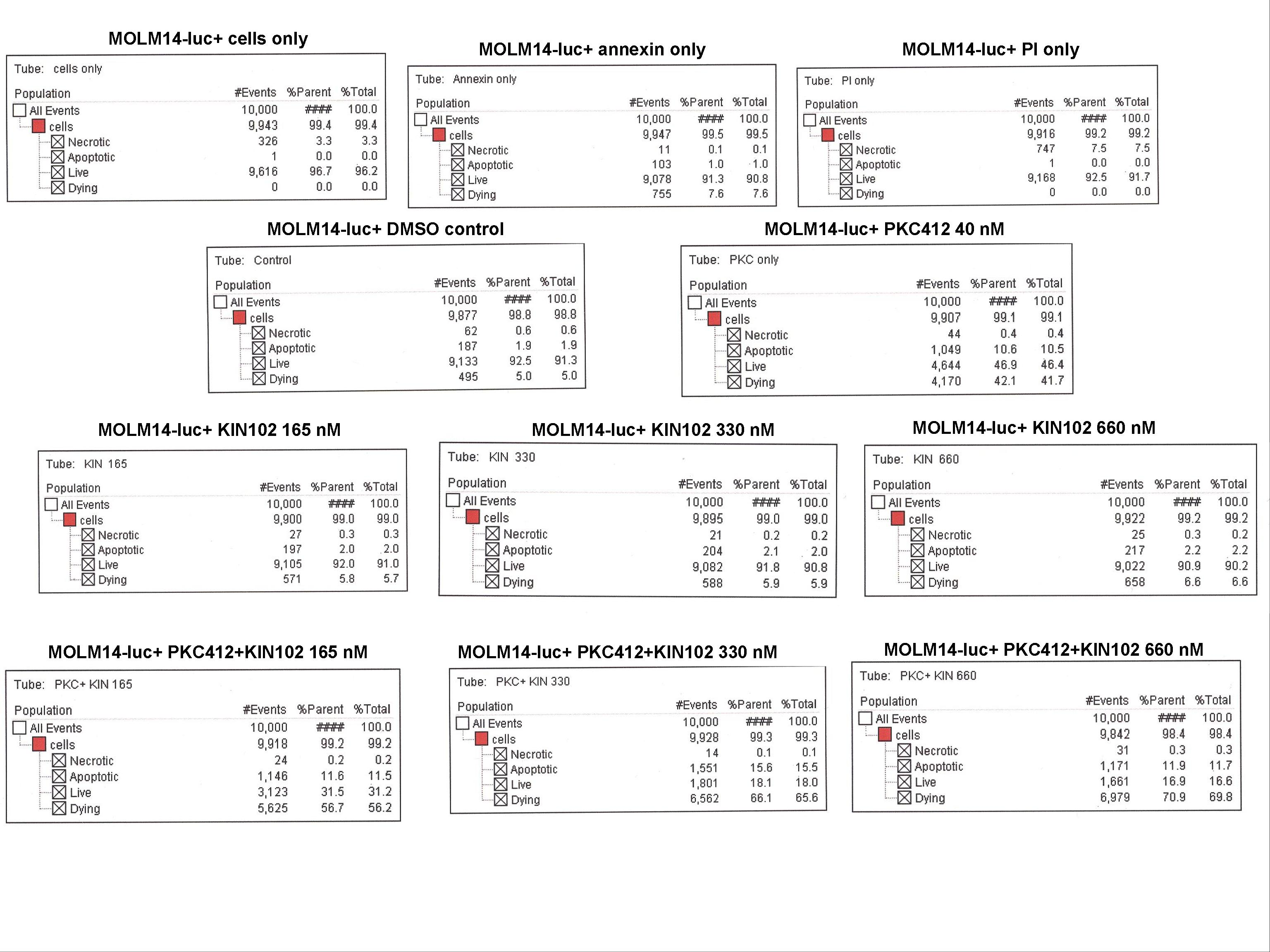


**Figure S7**

Supplement: Figure S7 — Part 1. Annexin/pi staining corresponding to data shown in Table 2: Effects of PKC412 (40 nM) and KIN001-102 (165, 330, 660 nM), alone and combined, on MOLM14-luc+ cell apoptosis (following 48 hours of treatment) when cells are cultured in the presence of RPMI+10% FBS. Cells labeled “dying” are in early apoptotic phase, and cells labeled “apoptotic” are in late apoptotic phase. Part 2. Quantitative values corresponding to data shown in Figure S7 (part 1): Effects of PKC412 (40 nM) and KIN001-102 (165, 330, 660 nM), alone and combined, on MOLM14-luc+ cell apoptosis (following 48 hours of treatment) when cells are cultured in the presence of RPMI+10% FBS. Cells labeled “dying” are in early apoptotic phase, and cells labeled “apoptotic” are in late apoptotic phase. (DOC) [file pone.0056473.s007.doc]

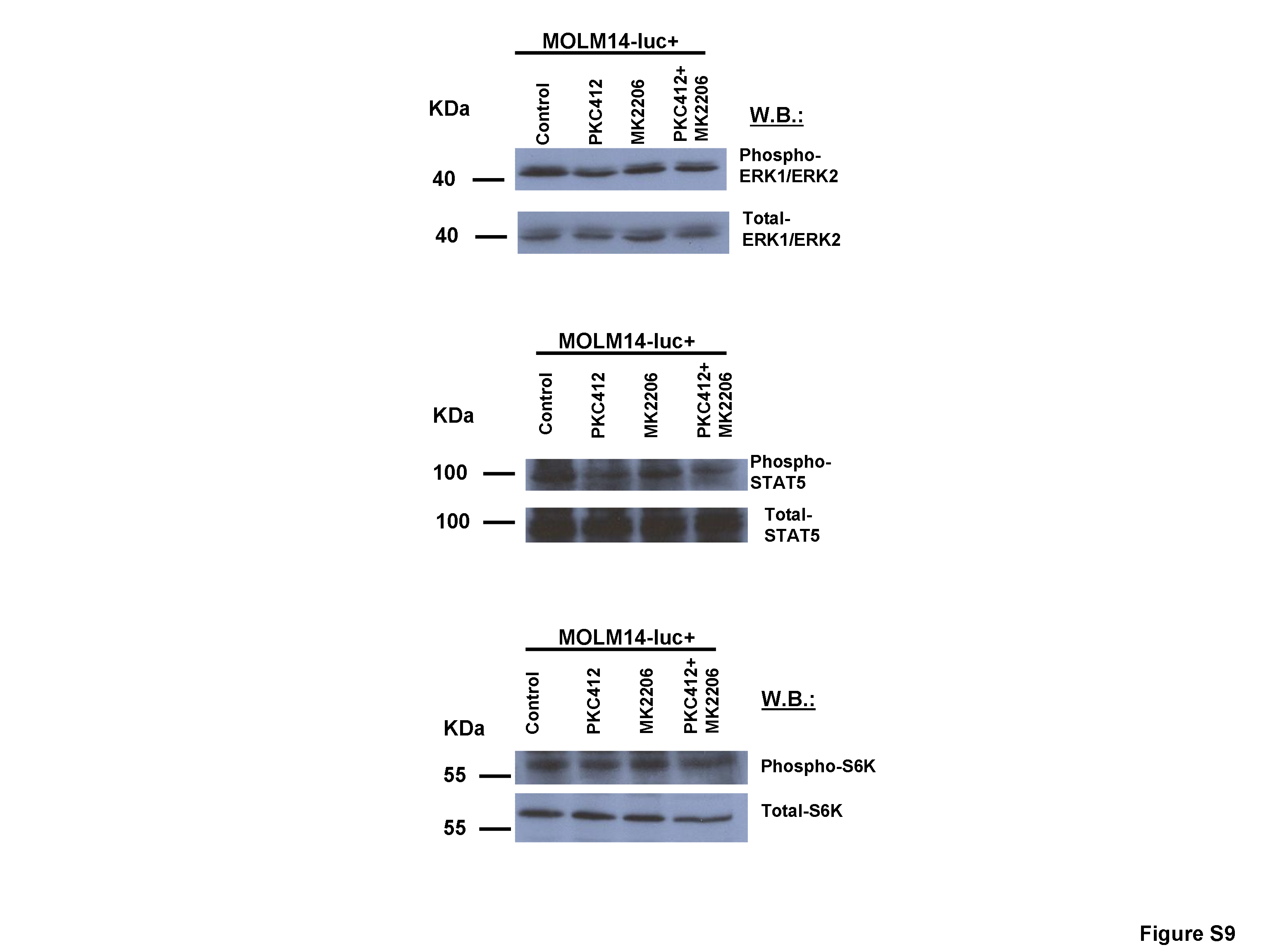

Supplement: Figure S9 — Investigation of phosphorylation of signaling molecules downstream of FLT3. Immunoblots of protein lysates prepared from MOLM14-luc+ cells treated for 1 hour with PKC412 (5 nM), MK2206 (165 nM), or a combination of the two agents in RPMI+10% FBS. (TIF) [file pone.0056473.s009.tif]
