# Supplementary material for: Selective Akt Inhibitors Synergize with Tyrosine Kinase Inhibitors and Effectively Override Stroma-Associated Cytoprotection of Mutant FLT3-Positive AML Cells
Source: PLoS One. 2013 Feb 21;8(2):e56473. doi: 10.1371/journal.pone.0056473 (PMC3578845; doi:10.1371/journal.pone.0056473)
Supplement: Table S1 — Patient sample information. Patients shown here were cultured in the presence of 50% HS-5 SCM, and treated with different combinations of kinase inhibitors. *Patient information for AML patients 2 and 7 has been previously published (Weisberg et al, 2012a, Leukemia). (DOC) [file pone.0056473.s010.doc]

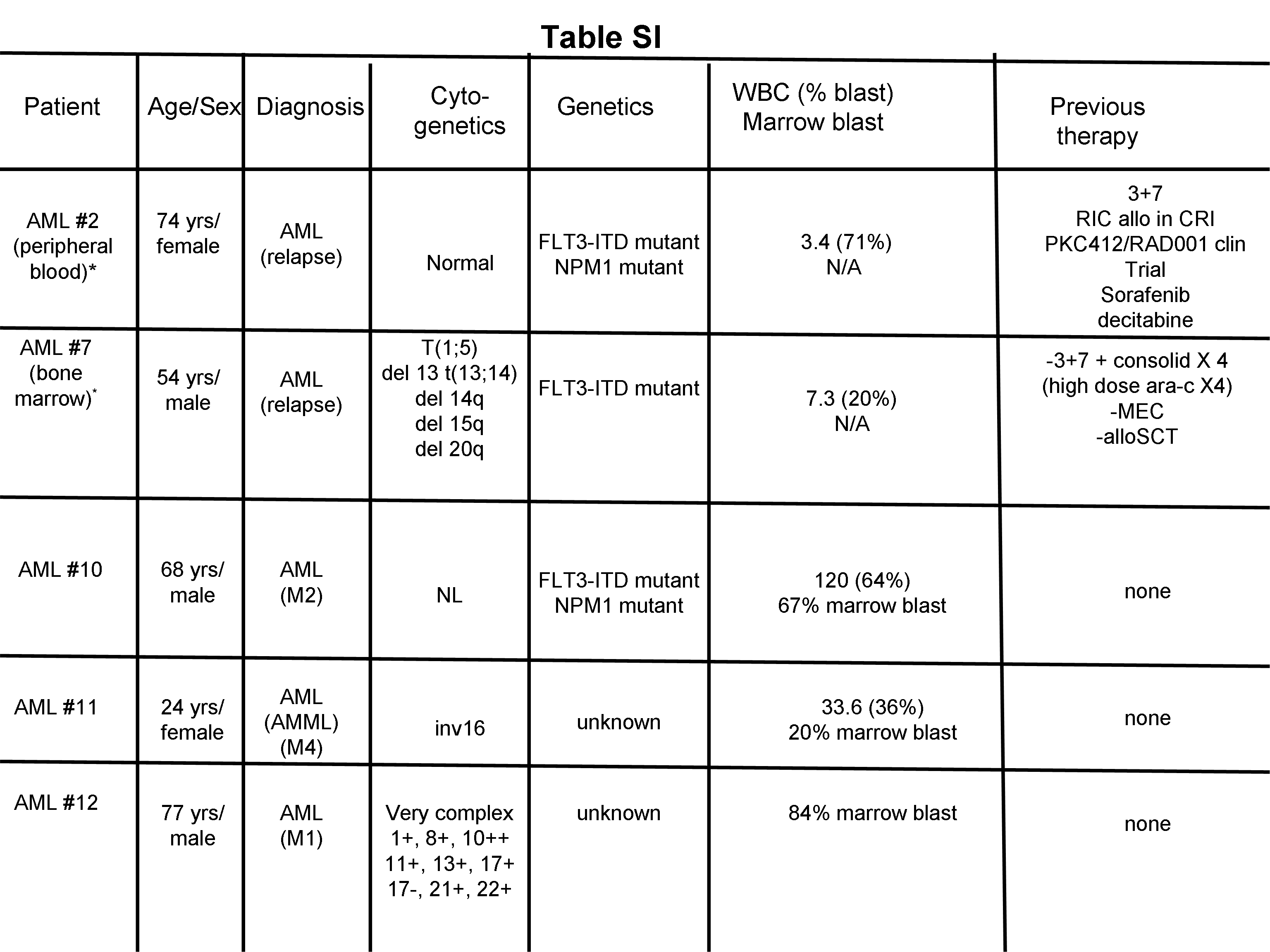


**Table S1. Patient sample information.** Patients shown here were cultured in the presence of 50% HS-5 SCM, and treated with different combinations of kinase inhibitors. *Patient information for AML patients 2 and 7 has been previously published (Weisberg et al, 2012a, Leukemia).
