# Supplementary material for: Selective Akt Inhibitors Synergize with Tyrosine Kinase Inhibitors and Effectively Override Stroma-Associated Cytoprotection of Mutant FLT3-Positive AML Cells
Source: PLoS One. 2013 Feb 21;8(2):e56473. doi: 10.1371/journal.pone.0056473 (PMC3578845; doi:10.1371/journal.pone.0056473)
Supplement: Table S2 — Selective AKT and p38 MAPK inhibitors. *Hirai H, Soontome H, Nakatsuru Y, Miyama K, Taguchi S, Tsujioka K et al. MK-2206, an allosteric Akt inhibitor, enhances antitumor efficacy by standard chemotherapeutic agents or molecular targeted drugs in vitro and in vivo. Mol Cancer Ther 2010;9∶1956-67. **Levy DS, Kahana JA, Kumar R. AKT inhibitor, GSK690693, induces growth inhibition and apoptosis in acute lymphoblastic leukemia cell lines. Blood 2009;113∶1723-9. ***Grimshaw KM, Hunter LJ, Yap TA, Heaton SP, Walton MI, Woodhead SJ, et al. AT7867 is a potent and oral inhibitor of AKT and p70 S6 kinase that induces pharmacodynamic changes and inhibits human tumor xenograft growth. Mol Cancer Ther 2010;9∶1100-10. (DOC) [file pone.0056473.s011.doc]

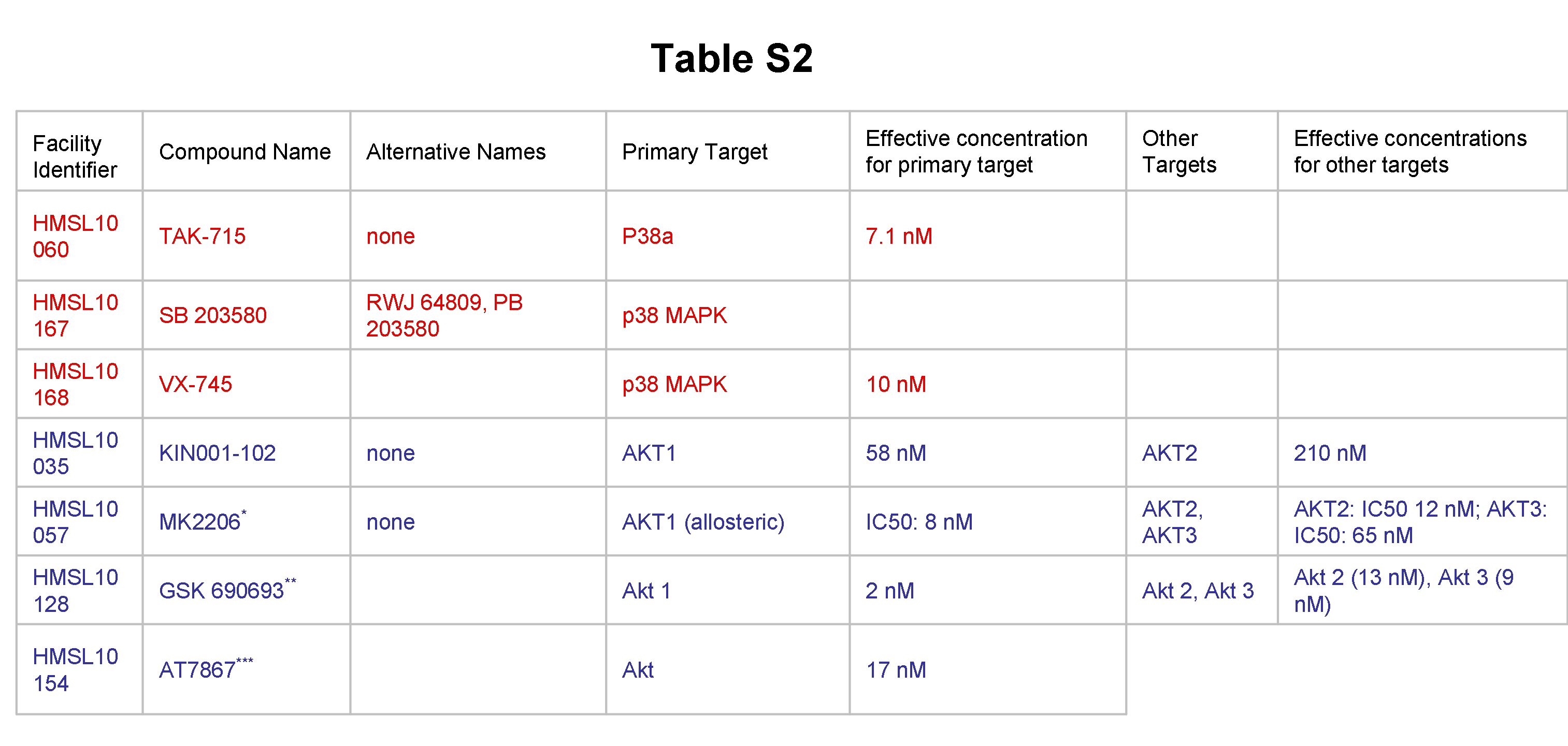


**Table S2. Selective AKT and p38 MAPK inhibitors.** *Hirai H, Soontome H, Nakatsuru Y, Miyama K, Taguchi S, Tsujioka K et al. MK-2206, an allosteric Akt inhibitor, enhances antitumor efficacy by standard chemotherapeutic agents or molecular targeted drugs in vitro and in vivo. Mol Cancer Ther 2010;9:1956-67. **Levy DS, Kahana JA, Kumar R. AKT inhibitor, GSK690693, induces growth inhibition and apoptosis in acute lymphoblastic leukemia cell lines. Blood 2009;113:1723-9. ***Grimshaw KM, Hunter LJ, Yap TA, Heaton SP, Walton MI, Woodhead SJ, et al. AT7867 is a potent and oral inhibitor of AKT and p70 S6 kinase that induces pharmacodynamic changes and inhibits human tumor xenograft growth. Mol Cancer Ther 2010;9:1100-10.
